# A splicing regulator, SR45, suppresses plant immunity by regulating salicylic acid pathway in *Arabidopsis thaliana*

**DOI:** 10.3389/fpls.2025.1704701

**Published:** 2025-10-31

**Authors:** Audrey Bui, Arden Bui, Iesh Gujral, Serena Fan, Anthony Long, Anna Hu, Christopher Chin, Jordan Powers, Ronghui Yang, Min Gao, Chong Zhang, Hua Lu, Bret Cooper, Xiao-Ning Zhang

**Affiliations:** ^1^ Department of Biology, St Bonaventure University, St Bonaventure, NY, United States; ^2^ Biochemistry Program, Department of Biology, St Bonaventure University, St Bonaventure, NY, United States; ^3^ Bronx High School of Science, Bronx, NY, United States; ^4^ Soybean Genomics and Improvement Laboratory, USDA-ARS, Beltsville, MD, United States; ^5^ Department of Biological Sciences, University of Maryland Baltimore County, Baltimore, MD, United States

**Keywords:** *Arabidopis thaliana*, metabolomics, transcriptomic (RNA-seq), alternative splicing (AS), salicylic acid, SR45, gene regulation and expression

## Abstract

Facing constant challenges from various pathogens and pests, plants have evolved different strategies to defend themselves both locally and systemically. A global change in RNA metabolism is one of the necessary steps to mount a long-lasting immunity against present and future invasions. *Arabidopsis* serine/arginine-rich 45 (SR45) is an evolutionarily conserved RNA-binding protein that regulates multiple steps of RNA metabolism. Our prior study suggested that SR45 acts as a negative regulator of plant immunity. To better understand the molecular mechanism for SR45’s defense role, we examined the metabolic profile in both Col-0 and *sr45-1*. The results showed a significant accumulation of pipecolic acid (Pip), salicylic acid (SA), and other potential defense compounds in *sr45-1*, indicating an increased systemic immunity. The *sr45–1* mutant exhibited an elevated resistance to a wide range of biotrophic pathogen species and insensitivity to Pip, SA, and pathogen pretreatment. Between the two alternatively spliced isoforms, SR45.1 and SR45.2, SR45.1 seemed to be the culprit for the observed immune suppression. Upon examination of the transcriptome profile between Col-0 and *sr45-1* under either mock or *Pseudomonas syringae Pma*DG3 challenge, we identified 1,125 genes as SR45-suppressed and *Pma*DG3-induced. Genes that function in SA biosynthesis and systemic acquired resistance were overrepresented, including those coding for WRKY, receptor-like kinases (RLKs), receptor-like proteins (RLPs), protein kinases, and TIR-NBS-LRR proteins. In addition, we identified significant alternative splicing activity in a list of genes due to either *sr45–1* alone or both *sr45–1* and *Pma*DG3 challenge. Among them, we characterized the effect of alternative splicing in two candidates, *CBRLK1* and *SRF1*. Interestingly, alternative splicing in both exhibited a switch between RLPs and RLKs in the predicted protein products. Overexpressing their *sr45–1* dominant isoform in Col-0 led to a partial increase in immunity, suggesting the involvement of both alternative splicing events in SR45-conferred immune suppression. In summary, we hypothesize that SR45 regulates a subset of immune genes at either transcriptional or co-transcriptional pre-mRNA splicing levels to confer its function in systemic immune suppression.

## Introduction

Pathogen-caused plant diseases threaten crop yield and global food security (Savary et al., 2019). Current climate change could exacerbate this threat by increasing the risk of disease outbreak (Singh et al., 2023). During evolution, plants have evolved sophisticated defense mechanisms to ward off pathogens. However, defense is an energy-demanding process, the activation of which could be at the expense of plant growth and development. Indeed, growing evidence has demonstrated that plant defense is complex and negotiated with plant growth, development, and crosstalk with other physiological processes (Denance et al., 2013; Zhou et al., 2015; Dressano et al., 2020; Fanara et al., 2022; Fabian et al., 2023). Upon pathogen exposure, plants activate pathogen-associated molecular pattern (PAMP)-triggered immunity (PTI) (Dodds and Rathjen, 2010) and effector-triggered immunity (ETI) to defend themselves against local infections (Jones and Dangl, 2006). ETI can also lead to systemic acquired resistance (SAR) in plants to defend against a broad spectrum of pathogens at the whole plant level (Durrant and Dong, 2004). A non-protein amino acid, pipecolic acid (Pip), and a small plant hormone, salicylic acid (SA), are irreplaceable regulators for inducing transcriptional reprogramming in SAR. Pip-triggered immune responses partly work through SA signaling via NON-EXPRESSOR OF PATHOGENESIS-RELATED GENES1 (NPR1) (Bernsdorff et al., 2016). Upon sensing the presence of a pathogen, NPR1 shuttles from the cytosol to the nucleus to activate the expression of different defense genes (Kinkema et al., 2000).

Despite the existing understanding of how known immunity regulators function in defense responses, knowledge is fragmented on the function of these regulators at the molecular level. Emerging evidence in recent years has suggested that pre-mRNA alternative splicing is an essential part of plant defense (Howard et al., 2013; Yang et al., 2014; Kufel et al., 2022; Fabian et al., 2023). For instance, many plant resistance (R) genes [such as *SNC1*, *RPS4*, and *RPS6* in *Arabidopsis*; *N* in tobacco; *BS4* in tomato; *RCT1* in *Medicago*; and *Y-1* in potato] are alternatively spliced (Gassmann et al., 1999; Schornack et al., 2004; Yang et al., 2008; Kim et al., 2009; Zhu et al., 2010). In addition, spliceosome-associated proteins were found in the protein complexes with defense proteins, such as MOS4 and MOS12 (Xu, Xu et al., 2012). However, questions remain as to whether the alternative splicing machinery is directly involved in plant innate immunity and, if so, how alternatively spliced transcripts function differently to regulate plant defense.

RNPS1 is an evolutionarily conserved eukaryotic splicing regulator (Mayeda et al., 1999). Serine/arginine-rich 45 (SR45) is the *Arabidopsis* ortholog of RNPS1 (Zhang and Mount, 2009). Our previous work suggested that SR45 is a scaffold RNA-binding protein that helps locate nearby splice sites and that SR45 and its interactors work together to fine-tune splicing (Zhang et al., 2017). In the SR45 interactor network, the exon junction complex (EJC) examines the quality of mRNAs before they are used as templates for translation (Fanara et al., 2024). A recent study in human cells found that nonsense-mediated decay largely relies on RNPS1 (Mabin et al., 2018). This makes RNPS1 a gateway to couple alternative splicing with the RNA quality control machinery. In addition, RNPS1 is a part of the apoptosis and splicing-associated protein (ASAP) complex, which recruits histone deacetylases, such as HDA19, to the affected locus and causes transcriptional silencing (Schwerk et al., 2003; Questa et al., 2016). The multifaceted involvement of RNPS1 in transcriptional regulation, splicing, and RNA quality control denotes its significance in the regulatory network for RNA metabolism. Moreover, the pre-mRNA of *SR45* is alternatively spliced, which results in the translation of two different protein isoforms, SR45.1 and SR45.2. These two isoforms play distinct roles during plant growth and development (Zhang and Mount, 2009).

Consistent with the notion that alternative splicing is important for plant growth and development, a null *Arabidopsis* mutant, *sr45-1*, exhibits growth and developmental defects, including delayed root growth, narrower leaves and flower petals, late flowering, unusual numbers of floral organs, iron deficiency, and mild sterility (Ali et al., 2007; Zhang et al., 2017; Fanara et al., 2022). In addition, the *sr45–1* mutant has altered sensitivity to both abiotic and biotic stimuli (Carvalho et al., 2010; Zhang et al., 2017; Albaqami et al., 2019). In our previous study, we observed that the *sr45–1* mutant exhibited an elevated PTI in responses to flg22 and a virulent strain of *Pseudomonas syringae Pma*DG3. In addition, we detected an elevated level of salicylic acid in the *sr45–1* mutant. Transcriptome analysis from the inflorescence tissue showed that SR45 preferentially suppressed immunity genes (Zhang et al., 2017). This led to the hypothesis that SR45 is a negative regulator of plant innate immunity. However, reproductive tissue and vegetative tissue respond to biotic stress differently. Since most pathogen infections occur in the vegetative stage of the plant cycle, in this study, we decided to examine how SR45 affects immunity in *Arabidopsis* 24-day-old leaves.

In this study, we attempted to elucidate the defense role that SR45 plays. We provide evidence here that the *sr45–1* mutation is responsible for the autoimmunity detected in the null mutant. The *sr45–1* mutant has an inherently higher SAR without pathogen priming, which offers the mutant plant a higher resistance to broad-spectrum biotrophic pathogens. This elevated broad-spectrum resistance is SR45.1-dependent. In wild-type plants, SR45 suppresses SAR by keeping Pip and SA levels low, downregulating SA pathway genes and downstream SA-responsive genes, and promoting the less defense-responsive splicing pattern of some defense genes. Together, our findings suggest that SR45 suppresses multiple aspects of the SA pathway via transcriptional and co-transcriptional controls to keep plant immunity under control during plant vegetative growth and development.

## Material and methods

### Plant growth conditions

All *Arabidopsis thaliana* plants used in this study were derived from the *Columbia* (Col-0) background. Mutant plants were either from the Arabidopsis Biological Resource Center (ABRC) or maintained in the lab. The primers used to confirm genotypes are listed in **Supplementary Table S1**. All plants were grown in conditions previously described (Zhang et al., 2017). The plants used in the defense assay were grown in soil with an L/D = 12 h/12 h photoperiod and 60% humidity.

### Cloning

The *SR45* promoter and *SR45* gene were amplified as a 3-kb fragment from Col-0 genomic DNA, digested with restriction enzymes *Not*I and *Hin*dIII, and then cloned into a modified expression vector *pGlobug-mCherry*, where the *GFP* coding sequence was replaced with *mCherry* using restriction enzymes *Hin*dIII and *Eco*RI. The entire expression cassette (*SR45 promoter::SR45 gene::mCherry::NOSTer*) was subcloned into a binary vector *pMLBartK* using *Not*I. *pMLBartK* replaced the Basta resistance gene in *pMLBart* with the kanamycin resistance gene for the transgenic plant screen.

*CBRLK1.2*, *CBRLK1.10*, and *SRF1.5* were amplified from cDNAs and subcloned into *pGlobug* (Chen et al., 2019). Then, the entire expression cassettes (*35S promoter::CBRLK1.2/CBRLK1.10/SRF1.5::NOSTer*) were subcloned into pMLBart using *Not*I.

### Generation of transgenic plants

The *pBartK-pSR45::SR45::mCherry::NOSTer* and *pBart-35S::CBRLK1.2/CBRLK1.10/SRF1.5-GFP::NOSTer* plasmids were introduced into *Agrobacterium tumefaciens* GV3101. The resulting Agrobacteria were used to transform *Arabidopsis* plants. Transgenic *Arabidopsis* plants for *SR45pro::SR45-mCherry* were selected on 1/2 Murashige and Skoog (MS) agar medium containing 50 mg L^−1^ kanamycin. Transgenic *Arabidopsis* plants for *35S::CBRLK1.2/CBRLK1.10/SRF1.5* were selected using 1:1,000 diluted Finale.

### RNA isolation, rtPCR, and rt-qPCR

RNeasy Mini Plus Kit (Qiagen, Germantown, MD) was used to isolate total RNA. Five micrograms of total RNA per sample was treated with DNase (Thermo Fisher) and then used for reverse transcription using SuperScript IV Reverse Transcriptase (Thermo Fisher, Grand Island, NY). The resulting cDNA was used for either alternative splicing PCR using Phusion PCR Mix (NEB, Ipswich, MA) or real-time quantitative PCR (qPCR) on CFX Connect qPCR Detection System (Bio-Rad Inc., Hercules) using SYBR Green PowerUp Master Mix (Thermo Fisher, Grand Island, NY). *GAPDH* was used to normalize results. The primers are listed in **Supplementary Table S1**.

### Library preparation and Illumina sequencing

Three biological replicates were used for each of the four samples: Col-0_Mock, Col-0_DG3, *sr45-1_*Mock, and *sr45-1_*DG3. A total of 2.5–5 μg of total RNA was used per sample for Illumina RNA-seq library preparation using TruSeq Stranded mRNA Library Prep Kit (Illumina, Inc., San Diego, CA). IDT unique dual indexes (UDIs) were used for multiplexing. High-throughput sequencing was performed using Nova-Seq 6000 (CD Genomics, Shirley, NY).

### RNA-seq data analysis

The quality of.fastq files was examined using FastQC (0.11.9) (Andrews). Trimmomatic (Bolger et al., 2014) was used to remove the first 10 nt from reads. The trimmed high-quality.fastq data were aligned to the *Arabidopsis* transcriptome AtRTD3 (Zhang et al., 2022) using *Salmon* (1.3.0) (Patro et al., 2017) to calculate transcript abundance in transcripts per million (TPM) and *p*-values. All these operations were performed on Galaxy. Then, differentially expressed (DE) genes, differentially alternatively spliced (DAS) genes, and isoform switch (IS) events were identified using the 3D RNA-seq App (Guo et al., 2021). Gene lists of interest were sent to Protein Analysis Through Evolutionary Relationships (PANTHER 16.0) for Gene Ontology (GO) enrichment analysis (Mi et al., 2021).

### Protein modeling and alignment

Deduced amino acid sequences for proteins of interest were obtained using ORFFinder (NCBI) using transcript sequences from the AtRTD3 transcriptome reference. These amino acid sequences were submitted to I-Tasser (Yang et al., 2015) for protein modeling. Protein models with the highest confidence were used for structure alignment analysis in the PyMOL Molecular Graphics System, version 1.3 (Schrödinger, LLC).

### Metabolomics sample preparation, mass spectrometry, and data analysis

#### Sample preparation

Total metabolites were extracted from 300 mg of fully expanded leaves of 27-day-old plants (W for Col-0 and *s* for *sr45-1*) with 1 mL of methanol in five biological replicates. An internal control of 5 μL of 250 pmol/μL prednisone was spiked into the methanol extract. All extracts were placed in a −20 °C freezer overnight. Two matrix blanks were prepared separately. Then, all extracts were centrifuged at 20,000 rpm at 4 °C for 30 minutes. Then, the clear supernatant was transferred to glass vials. All solvents were evaporated completely. The residues were stored in a −20 °C freezer till further analysis.

#### Mass spectrometry

The residues of the W and *s* replicates and two corresponding matrix blanks were separately resuspended in 120 µL 50% methanol/0.1% formic acid. Residual insoluble particulate matter was removed by centrifugation (12,000 × *g*, 20 minutes at 4 °C). Twenty-five microliters from each leaf sample was combined to make a quality control (QC) pool. Five-microliter injections of the samples, blanks, and QC were used in the subsequent procedure. Injections were separated on a 150 × 2.1 mm Hypersil GOLD VANQUISH HPLC column with 1.9 µm particles (Thermo Fisher Scientific) at 40 °C coupled to a Vanquish HPLC pump (Thermo Fisher Scientific) controlling a 10-minute linear gradient from 0% to 95% acetonitrile and 0.1% formic acid at a flow rate of 0.2 mL per minute. Eluent was electrosprayed at 3.5-kV positive polarity into an Exploris 240 mass spectrometer (Thermo Fisher Scientific). The instrument was calibrated externally at less than 1 ppm root mean square (RMS) deviation in the low and high mass ranges and dynamically calibrated using an internal mass calibrant during MS1 scanning. Sheath gas was 35, auxiliary gas was 7, and sweep gas was 1 (arbitrary units). The ion transfer tube temperature was 325 °C, and the vaporizer temperature was 275 °C. The default charge state was 1, and the expected peak width was 6 sec. Advanced peak determination, mild trapping, internal mass calibration, and the AcquireX method modifications were enabled. AcquireX Deep Scan was used to create a background ion exclusion list from the matrix blank and an inclusion ion list from the QC. Default deep scan settings were used except that [M+H]+1 ions were preferred and isotopes of fragmented precursors were excluded. MS1 survey scans were recorded in the Orbitrap at 120,000 resolution over a mass range of 70–800 *m/z*. The radio frequency (RF) lens was 70%, the automatic gain control (AGC) target was standard, the maximum injection time was 100 milliseconds, and the microscan was 1. MS2 analysis was not performed at this step. After the matrix blank injection to create an exclusion list and the QC injection to create an inclusion list, five injections of the QC were performed using AcquireX to generate MS2 spectra for identification (ID). MS1 survey scans were recorded in the Orbitrap at 60,000 resolution over a mass range of 70–800 *m/z* using the prior settings for MS1 scanning. Additionally, monoisotopic precursor selection was enabled, the minimum intensity was 5,000, charge states were filtered to 1, dynamic exclusion was set at auto, and target mass and targeted mass exclusions had 3-ppm mass windows. Twenty precursor ions per cycle were selected within a 1.0-Da isolation window and were fragmented by high-energy collision-induced dissociation (30%, 50%, and 70% normalized stepped collision energy), and their MS2 fragment ions were resolved in the Orbitrap at 30,000 resolution with a standard AGC target, maximum injection time of 54 milliseconds, and 1 microscan. After each ID injection, the *m/z* of the resolved ions was automatically appended to the exclusion list for the subsequent injection. When the ID injections were completed, the samples were injected in the following order: matrix blank 1, QC1, W1, *s*1, matrix blank 2, QC2, W2, *s*2, etc. MS1 survey scans were recorded in the Orbitrap at 120,000 resolution over a mass range of 70–800 *m/z*. The RF lens was 70%. MS2 analysis was not performed for these replicates and matrix blanks. This entire procedure was repeated using the same samples but with the mass spectrometer operating in negative ion mode at −2,500 V. The other settings were the same except that the AcquireX preferred ion was [M−H]−1.

#### Compound discoverer data analysis

Positive ion mode and negative ion mode mass spectrometry data files were analyzed separately using Compound Discoverer version 3.3 (Thermo Fisher Scientific). The Input File node was used to submit the 10 files for the biological injections, the corresponding matrix blank and QC files, and the five corresponding ID files (but not the two files used for generating the initial exclusion and inclusion lists for the IDs). The Select Spectra node was used with open settings except for a 1.5 S/N threshold. The ChromAlign node was used to align chromatographic peaks in all files to the QC3 (or QC3neg) file. The Detect Compounds node was used with 2-ppm mass tolerance, 10,000 minimum peak intensity, at least five scans per peak, peak detection S/N threshold 1.5, gap ratio threshold 0.35, max peak width 0.25, and compound detection of [M+H]+1 ions for positive ion mode and [M−H]−1 for negative ion mode. The Group Compounds node was used with 2-ppm mass tolerance, 0.25-minute retention time (RT) tolerance, peak alignment true, and a peak rating filter threshold of 4 for a minimum of six files. The Fill Gaps node was used with 2-ppm mass tolerance; the SERRF QC Correction node was used with 80% QC coverage to normalize the peak area results, max QC area RSD 30%, max corrected QC area RSD 25%, and correct blank files true; and the Mark Background Components node was enabled with max sample/blank 3. The Search mzCloud node was used to compare MS2 spectra from ID files with the cosine identity algorithm to all compound classes at a precursor mass tolerance of 5 ppm, fragment mass tolerance of 5 ppm, and no other filters. The Search mzVault node was used to compare MS2 spectra from ID files to the NIST_2020_MSMS High Resolution library and to a custom library of 20 amino acid standards using FT precursor and fragment mass tolerances of 10 ppm with the HighRes algorithm and no other filters. The Predict Compositions node was used at 2-ppm mass tolerance with element counts C90 H190 N10 O18 P5 S5. The Apply mzLogic and Apply Spectral Distance nodes were used with 2-ppm mass tolerances. Filtering of results was performed to limit background ions, include normalized areas, and require MS2 of preferred ions. The software performed a two-tailed Student’s t-test to estimate compound peak area statistical differences and used the Benjamini and Hochberg method to estimate the false discovery rate (FDR). Filtering of results was performed to limit background ions, for normalized peak areas, and to require MS^2^ supporting spectra.

### Plant defense assays

For testing basal defense, assays were performed using a previously described protocol with modification (Greenberg and Ausubel, 1993). A virulent strain of *P. syringae* pv. *maculicola* DG3 (*Pma*DG3, a *recA* derivative of ES4326) and two avirulent strains, *Pma avrRpt2* (DG6) and *Pma avrRpm1* (DG34), were inoculated in King’s Broth (KB) overnight at 30 °C. The overnight culture was then refreshed with a 1:5 dilution in KB for 5 h. A bacterial density of OD_600_ = 0.0001 for DG3 or OD_600_ = 0.0004 for DG6 and DG34 in 10 mM MgSO_4_ was used to infiltrate leaves 4–6 of 24-day-old *Arabidopsis* plants. Three days later, 4-mm-diameter leaf discs were punched, ground, diluted, and plated on KB medium containing 50 mg L^−1^ kanamycin for bacterial colony count.

For oomycete treatment, the *Hyaloperonospora arabidopsidis* (*Hpa*) Noco2 virulent strain was prepared and sprayed as previously described (Zhang et al., 2017).

For determining SAR, leaves 3 and 4 of 3-week-old plants were infiltrated with either 10 mM MgSO_4_ (mock) or *Pma*DG6 suspension (OD_600_ = 0.01). Then, leaves 5–7 were infiltrated with *Pma*DG3 (OD_600_ = 0.001) 3 days later. After three more days, 4-mm-diameter leaf discs were punched for bacterial colony count as described above.

#### Exogenous application of SA and Pip

Three-week-old plants were sprayed with either sterilized water or 0.5 mM SA. For pipecolic acid application, 10 mL of sterilized water or 10 mL of 1 mM pipecolic acid solution was pipetted onto the soil surrounding each plant. One day later, leaves 3–6 were infiltrated with *Pma*DG3 (OD_600_ = 0.001). After three more days, 4-mm-diameter leaf discs were punched for bacterial colony count as described above.

### Statistical analyses

A Shapiro–Wilk normality test was conducted to assess sample distribution. For samples that passed the normality test, one-way ANOVA followed by Tukey’s Honestly Significant Difference (HSD) test was used for statistical analysis. For samples that did not pass the normality test, the Kruskal–Wallis test followed by Dunn’s test was performed to determine statistically significant differences. The Benjamini–Hochberg method was used to estimate FDR for high-throughput sequencing results.

## Results

### SR45-mCherry rescued pleiotropic phenotypes in the sr45–1 mutant

Our previous study showed that the two alternatively spliced isoforms of SR45 (SR45.1 and SR45.2) exhibited distinct functions in plant development and growth (Zhang and Mount, 2009). To account for the contributions of both isoforms, we introduced a native copy of the *SR45* gene fused with *mCherry* driven by the *SR45* native promoter into the *sr45–1* null mutant. More than 20 independent transgenic lines showed a strong mCherry signal in various tissues, such as pollens, ovules, cotyledons, and root tips (**Supplementary Figures S1A–D**). We selected three lines, #1, #3, and #9, for further analysis. All these three lines expressed *SR45* transcripts to a level similar or slightly higher than that of Col-0 (**Supplementary Figure S1E**), while they all expressed a wild-type (WT) level of *FLC* transcripts (**Supplementary Figure S1F**). All three transgenic lines resembled WT Col-0 in many aspects, including plant size, flowering time, root growth, and immune response to *P. syringae* (**Supplementary Figure S1G–J**). Taken together, we concluded that the transgene *SR45-mCherry* was able to rescue the pleiotropic phenotypes of the *sr45–1* mutant. Specifically, SR45 functions as a *bona fide* suppressor of plant immunity. Out of the three transgenic lines, line #3 resembles the WT the best. Therefore, we used this line for all future experiments and refer to it as *SR45-mCherry* from now on.

### Defense compounds are overaccumulated in the sr45–1 mutant

**Figure 1:**
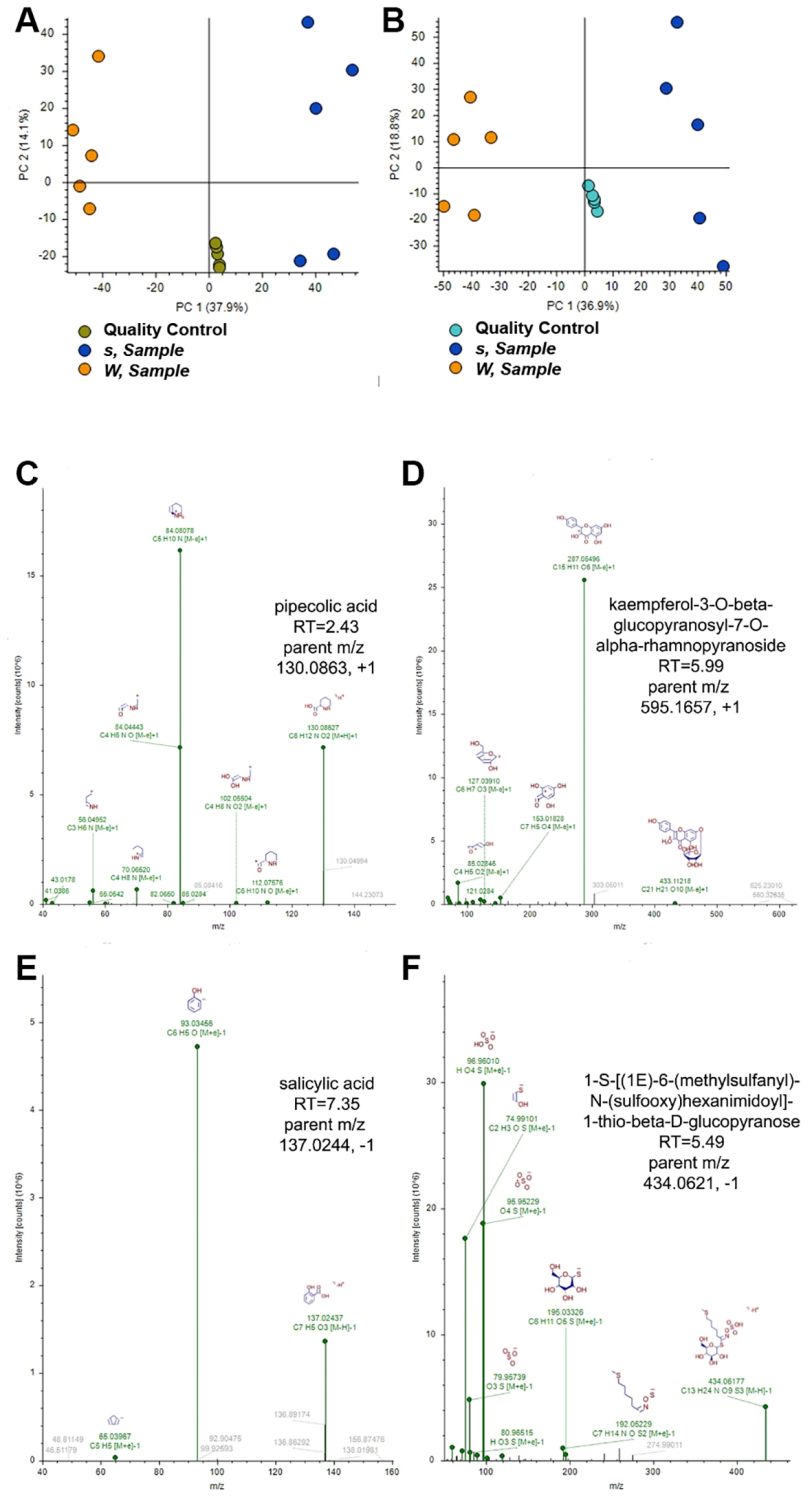
A summary of the metabolomics study between Col-0 and *sr45-1*. **(A, B)** Principal component analysis (PCA) scoring plots of quantified compounds from *sr45-1* (*s*) and control (W) leaves and quality control (QC) replicates (n = 5). **(A)** Positive ion mode compounds. **(B)** Negative ion mode compounds. **(C–F)** Annotated tandem mass spectra for compounds with increased abundance in *sr45-1*. **(C)** Pipecolic acid. **(D)** Kaempferol-3-*O*-β-glucopyranosyl-7-*O*-α-rhamnopyranoside. **(E)** Salicylic acid. **(F)** 1-*S*-[(1*E*)-6-(Methylsulfanyl)-*N*-(sulfooxy)hexanimidoyl]-1-thio-beta-d-glucopyranose.

In order to see if the *sr45–1* mutant produces a different metabolic profile from Col-0, all the metabolites in Col-0 and *sr45–1* mutant leaves were extracted from 4-week-old adult plants, and a metabolomics study was conducted. After subtracting background and non-qualifying peaks, a total of 3,772 compounds were identified in positive ion mode, and 3,187 were identified in negative ion mode. Principal component analysis confirmed that the *sr45–1* sample and control extracts were distinct in both positive and negative ion modes (**Figures 1A, B**). Filtering the compound results for FDR < 5% and |*sr45-1*/Col-0 (*s*/W) log2 fold-changes| ≥ 0.50 yielded 940 compounds in positive ion mode and 796 compounds in negative ion mode. Compounds with increased fold changes in *sr45–1* samples were further selected for mzCloud or NIST_2020_MSMS spectrum match scores ≥70. Results were examined by hand and discarded if the chromatographic peaks were substandard, if the MS^2^ identifying spectrum did not fall within the assigned chromatographic peak area, or if the match fell outside of the 2-ppm mass tolerance window. Select compounds with significantly increased abundance are shown in **Supplementary Table S2**.

Among the significantly increased compounds from *sr45–1* leaves detected in positive ion mode were Pip (*s*/W log2 fold-change = 2.37) (**Figure 1C**), three isomers of kaempferol-3-*O*-β-glucopyranosyl-7-*O*-α-rhamnopyranoside, and a glycosidic flavonoid (**Figure 1D**; *s*/W log2 fold-changes ranged from 1.13 to 0.52). Among the significantly increased compounds from *sr45–1* leaves detected in negative ion mode were SA (**Figure 1E**; *s*/W log2 fold-change = 1.28) and 1-S-[(1*E*)-6-(methylsulfanyl)-*N*-(sulfooxy)hexanimidoyl]-1-thio-beta-d-glucopyranose, a glucosinolate (**Figure 1F**; *s*/W log2 fold-change = 2.46). The tandem mass spectra and retention times for both Pip and SA were confirmed by comparison to purified standards analyzed under the same conditions. The higher SA level seen here in the *sr45–1* mutant is consistent with our previous report (Zhang et al., 2017).

Both Pip and SA are well-known defense hormones for SAR in plants. It has been proposed that SA-triggered SAR is located downstream of Pip (Bernsdorff et al., 2016). Having an abnormally high level of both Pip and SA could lead to a hyperactive SAR in the *sr45–1* mutant even without any pathogen challenges. Both kaempferol and glucosinolates are known for their role in plant defense (Bednarek et al., 2009; Likić et al., 2014; Onkokesung et al., 2014). Therefore, the *sr45–1* mutant inherently exhibited a metabolic profile of a heightened defense without any pathogen challenges.

### SR45 regulates both local resistance to a broad spectrum of biotrophic pathogens in an isoform-specific manner and systemic acquired resistance

**Figure 2:**
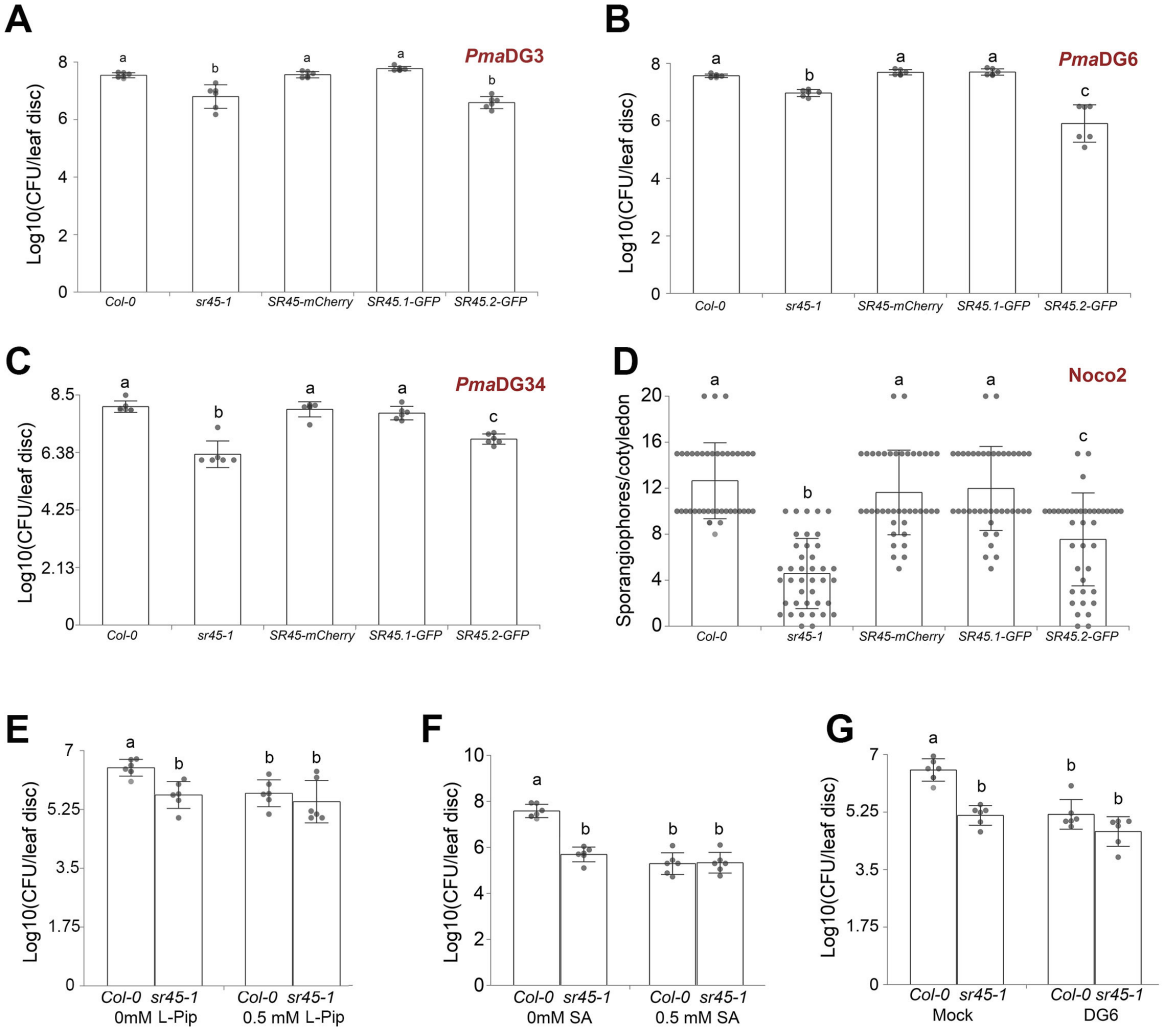
SR45 suppresses both local and systemic defense responses. One-way ANOVA followed by Tukey’s HSD test was used for statistical analysis. Error bars represent standard deviations. Letters a–c represent statistically significant difference (*p* < 0.05). *SR45-mCherry* represents *SR45pro::gSR45-mCherry sr45-1*. All experiments were repeated for at least two trials. **(A)** The virulent strain *Pma* DG3 was used for local response, n = 6. **(B)** The avirulent strain DG6 (*Pma avrRpt2*) was used for local response, n = 6. **(C)** The avirulent strain DG34 (*Pma avrRpm1*) was used for local response, n = 6. **(D)** The virulent strain *Hpa* Noco2 was used for local response, n = 40. **(E)** 0.5 mM of l-Pip treatment followed by DG3 was used for systemic response, n = 6. **(F)** 0.5 mM of SA treatment followed by DG3 was used for systemic response, n = 6. **(G)** DG6 treatment followed by DG3 was used for systemic response, n = 6. Pip, pipecolic acid; SA, salicylic acid.

To see if the elevated defense response in the *sr45–1* mutant is pathogen strain-specific, we infected the *sr45–1* mutant with either virulent or avirulent bacterial pathogens, *P. syringae* pv. *maculicola* (*Pma*)DG3, *Pma*DG6, and *Pma*DG34, and the virulent oomycete pathogen *H. arabidopsidis Noco2*. The *sr45–1* mutant exhibited an elevated immunity against all of them, and the *SR45-mCherry* transgene was able to fully recover the mutant immune response back to the WT level for all these pathogens (**Figures 2A–D**). Since *SR45* itself is alternatively spliced, the two alternatively spliced isoforms, *SR45.1* and *SR45.2*, exhibit distinct functions (Zhang and Mount, 2009). We examined which alternatively spliced isoform of *SR45* is responsible for its defense role. We treated WT, the *sr45–1* mutant, along with previously confirmed transgenic plants expressing only isoform 1 (*SR45.1-GFP*) or only isoform 2 (*SR45.2-GFP*), with the same set of pathogen species as mentioned earlier. The results consistently showed that *SR45.1*, but not *SR45.2*, was able to recover the mutant immune response to the WT level (**Figures 2A–D**), suggesting that *SR45* confers immune suppression to a broad spectrum of pathogens in an *SR45.1* isoform-dependent manner.

To see if the detected overaccumulation of Pip and SA in the *sr45–1* mutant would affect the immune response, we pretreated both Col-0 and *sr45–1* with Pip, SA, or *Pma*DG6 separately before exposing the plants to *Pma*DG3 challenge. All pretreatments were able to increase the resistance to *Pma*DG3 in Col-0 (**Figures 2E–G**). However, the *sr45–1* mutant readily exhibited a higher resistance under the mock condition at a comparable level as DG6-pretreated Col-0. Exogenous applications of Pip and SA, as well as *Pma*DG6 pretreatment, did not further increase the mutant’s resistance to *Pma*DG3 (**Figures 2E–G**). This corroborates the elevated defense metabolic profile in *sr45–1* that we identified earlier, further suggesting an inherent SAR response in the *sr45–1* mutant.

### SR45 suppresses plant immunity by regulating *Pma*DG3-induced genes

Plants activate extensive transcriptome reprogramming when facing pathogen challenges. To gain a transcriptome-level understanding of how a lack of SR45 could lead to a high level of Pip and SA and an elevated immunity, we examined the leaf transcriptome in Col-0 and *sr45–1* mutants under either mock treatment or 1 day after infection (DPI) by *Pma*DG3 infiltration.

**Figure 3:**
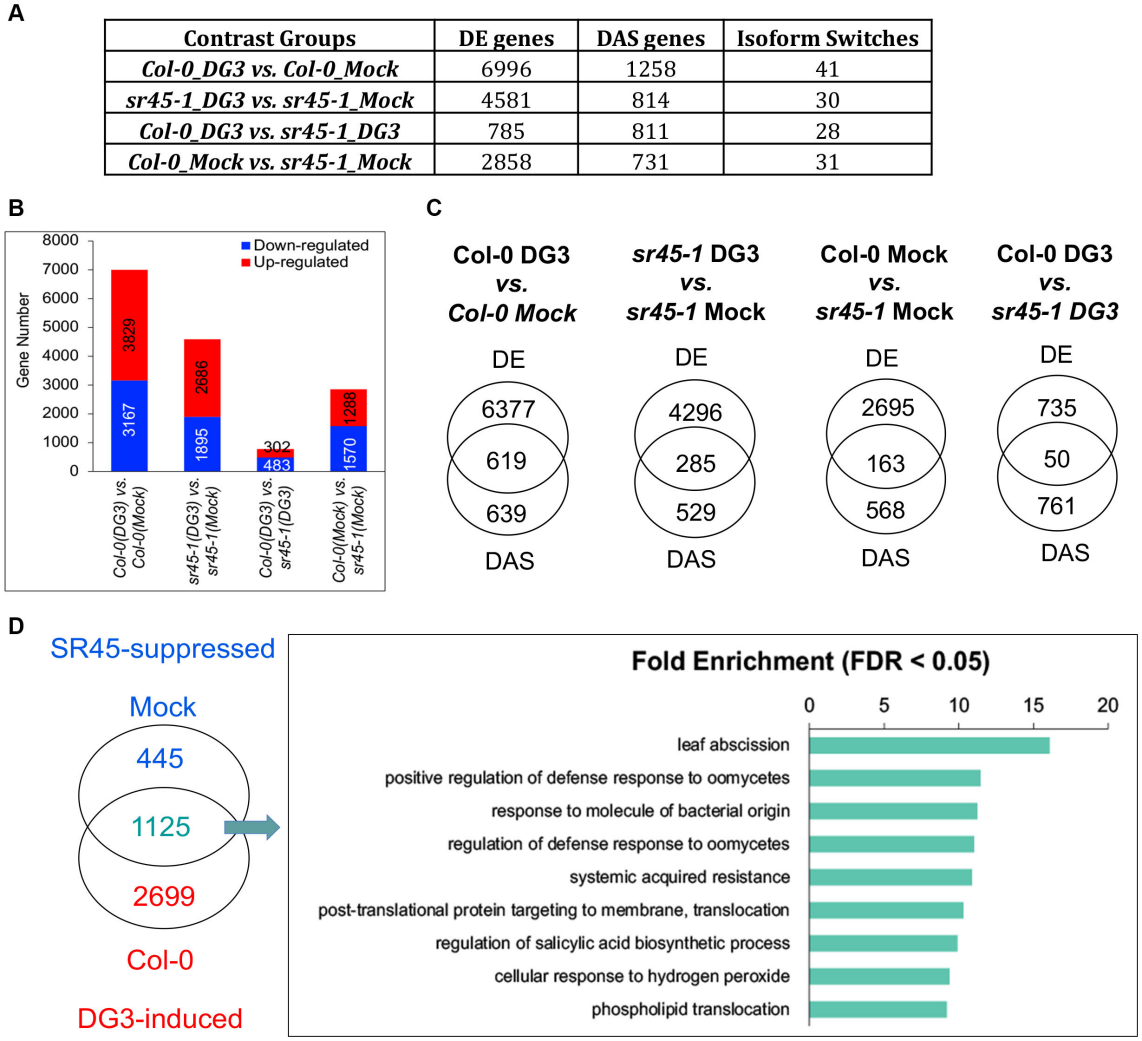
A summary of RNA-seq analysis results. **(A)** A comparison of gene numbers in different categories for each contrast group examined. **(B)** The number of upregulated and downregulated genes in each contrast group. **(C)** A comparison of DE and DAS genes in each contrast group. **(D)** A great overlap of SR45-suppressed genes under the mock condition and DG3-induced genes reported in wild type. GO enrichment analysis suggests that defense genes are overrepresented among these SR45-suppressed and DG3-induced genes. DE, differentially expressed; DAS, differentially alternatively spliced.

Transcriptome analyses returned a list of DE genes, DAS genes, and IS events for each comparison group, as shown in **Figure 3A**. When an alternatively spliced isoform pair exhibited a statistically significant switch in their abundance between two comparison groups, this was defined as an IS event. Based on these categories defined in 3D RNA-seq, we then asked the following questions: (1) How much of the differential expression was due to differential alternative splicing? (2) How many SR45-suppressed DE genes were also induced by pathogen challenge, and what are the molecular functions of these DG3-induced and SR45-suppressed genes? (3) Did alternative splicing make an impact at structural and possibly functional levels on any defense gene products?

### How much of the differential expression was due to differential alternative splicing?

*Pma*DG3 challenge led to more DE genes in Col-0 than in *sr45-1*. The smallest number of DE genes was identified in the Col-0_DG3 vs. *sr45-1*_DG3 contrast group (**Figure 3A**). There was no substantial difference between the number of upregulated genes (higher expression in the first sample, aka. induced genes, illustrated in red) and the number of downregulated genes (higher expression in the second sample, aka. suppressed genes, illustrated in blue) in each contrast group (**Figure 3B**). When comparing the identity of DE genes and DAS genes, it was observed that approximately 35%–49% of DAS genes, due to the DG3 challenge (619 in Col-0 and 285 in *sr45-1*), were also differentially expressed (**Figure 3C**). This indicates that a good proportion of DG3-induced alternative splicing events made a difference in the level of gene expression. However, only 6%–22% of SR45-dependent DAS genes (163 under mock and 50 under DG3 treatment) contributed to SR45-dependent differential expression (**Figure 3C**). Overall, the majority of DE and DAS genes are distinct in all contrast groups (**Figure 3C**). This could be due to the fact that alternative splicing is only one of the mechanisms to regulate transcriptome reprogramming, and a cascade of gene regulations was likely happening during the experiment. It is worth noting that less than 45% (499 out of 1,032) of the SR45-differentially regulated genes found in inflorescence from our previous study (Zhang et al., 2017) were identified as DE genes in leaf samples of the Col-0_Mock vs. *sr45-1*_Mock contrast group in this study. This difference could be due to tissue-specific gene expression and different analysis pipelines being used. Defense genes are highly overrepresented in the 449 common SR45 differentially regulated genes between leaf and inflorescence (**Supplementary Figure S2**; **Supplementary Table S3**), which further supports SR45’s role in regulating defense genes.

### How many SR45-suppressed DE genes were also induced by pathogen challenge, and what are the molecular functions of these DG3-induced and SR45-suppressed genes?

**Figure 4:**
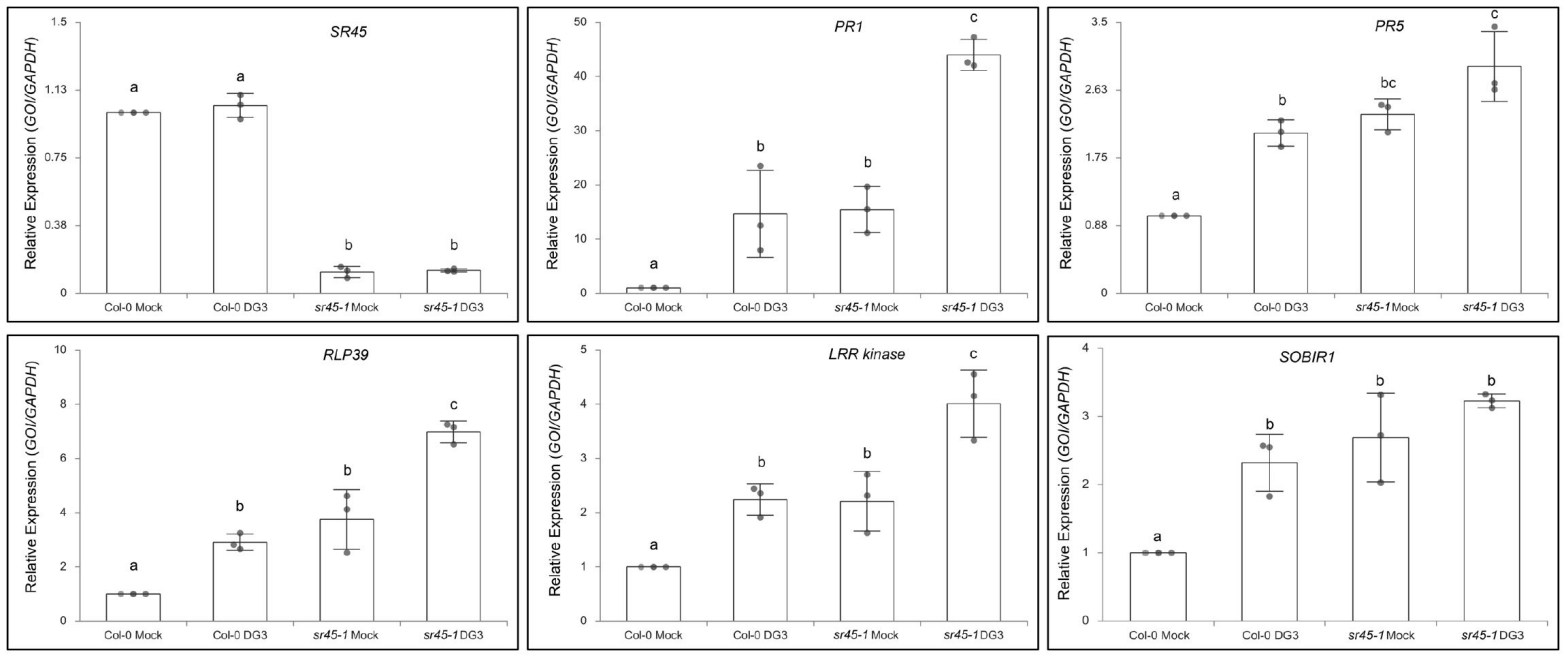
Real-time qPCR validation of selected SR45-suppressed and DG3-induced gene candidates found in RNA-seq analysis. *GAPDH* was used as the housekeeping control. n = 3. Error bars represent standard deviations. One-way ANOVA followed by Tukey's HSD test was used for statistical analysis. Letters a–c represent statistically significant difference (*p* < 0.05).

After comparing the identity of leaf DE genes in different contrast groups, we found the biggest overlap between the 1,570 SR45-suppressed genes under mock treatment and the 3,829 DG3-induced genes in Col-0. Approximately 72% (1,125/1,570) of the 1,570 SR45-suppressed genes under mock treatment were also within the 3,829 DG3-induced genes in Col-0. We defined these 1,125 overlapping genes as DG3-induced and SR45-suppressed. PANTHER GO Enrichment analysis reported that genes that function in systemic acquired resistance and regulation of SA biosynthesis, as well as other aspects of plant defense, are overrepresented in this new group (**Figure 3D**; **Supplementary Table S4**). The expression pattern of several genes of interest was further confirmed by qPCR as DG3-induced and SR45-suppressed, such as defense marker genes *PR1* and *PR5*, a receptor-like protein gene *RLP39*, an LRR kinase gene (*AT1G35710*), and *SOBIR1* (**Figure 4**). From this, we concluded that SR45 likely keeps a large number of defense genes at bay to minimize unwanted energy consumption when defense is not needed.

Since we detected an overaccumulation of Pip and SA in the *sr45–1* mutant earlier (**Figures 1C, E**), we looked for genes that code for Pip and SA synthesis and/or SA accumulation. Both *ALD1* and *SARD4* function in the Pip synthesis pathway (Ding et al., 2016). We identified the expression of both as DG3-induced and SR45-suppressed in this study. The SA biosynthesis/accumulation genes, e.g., *SID2*, *PAD4*, *EDS1*, *EDS5*, and *PBS3* (Mishra and Baek, 2021), were also DG3-induced and SR45-suppressed (**Supplementary Figure S3**; **Supplementary Table S4**). The combination of an increase in SA abundance from the metabolomics result and an increase in the expression of SA biosynthesis genes from the RNA-seq result suggests that it is very likely that SR45 regulates the SA level by controlling the expression of several SA biosynthesis genes. In addition, a higher SA level could lead to a more active SA signaling and increased expression of SA-induced genes to confer a systemic response in the *sr45–1* mutant. Some groups of defense genes are summarized below.

*WRKY transcription factors and their gene targets*: In SA-mediated SAR, defense-responsive WRKY transcription factors play a key role in promoting the expression of defense genes (Hussain et al., 2018). A total of 17 *WRKY*s were recognized among the 1,125 DG3-induced and SR45-suppressed genes. The protein products of most of them belong to Group II or Group III WRKY families (**Supplementary Table S4**). The well-known WRKY-binding sequence, *W-box* (*TTGACT*), was identified in the promoter region (−500 bp) of 76% (855/1,125) of the 1,125 DG3-induced and SR45-suppressed genes (**Supplementary Table S4**). This widespread pattern indicates that the majority of the DG3-induced and SR45-suppressed genes could be due to an indirect effect of WRKY transcription factors. However, we did not attempt to distinguish which ones were direct targets of WRKYs and which ones were not. These putative WRKY targets include quite a few receptor-like proteins (RLPs) and receptor-like kinases (RLKs).

To date, a total of 57 RLPs have been annotated in *Arabidopsis*, divided into two classes: 36 pathogen-induced and 21 basal RLPs (Steidele and Stam, 2021). In DG3-induced and SR45-suppressed genes, we found 18 *RLP* genes (**Supplementary Table S4**), all of which are categorized as defense-induced RLPs. We confirmed the expression pattern of one of them, *RLP39*, using qPCR (**Figure 4**).

*Kinases:* Genes coding for several types of RLKs, such as LRR-RLK, LecRK, CRK, and WAK, were found in DG3-induced and SR45-suppressed genes (**Supplementary Table S4**). *SOBIR1* was a DG3-induced and SR45-suppressed *LRR-RLK*. It has been shown that SOBIR1 protein interacts with RLP23, a confirmed defense-responsive LRR-RLP, to recruit BAK1 and trigger downstream signaling for defense (Albert et al., 2015). In our experiment, neither *BAK1* nor *RLP23* was both DG3-induced and SR45-suppressed at the RNA level. This makes *SOBIR1* an important target in SR45-conferred immune suppression. Mitogen-activated kinase cascade player genes, such as *MEKK3*, *MKK1*, *MKK2*, *MPK1*, and *MPK11*, were among those affected by SR45. Their translated products may function in the cytoplasm to relay signals perceived by membrane-bound RLKs and/or RLPs. An example is the *AT1G35710* locus that encodes such an LRR kinase. Mosher et al. showed that the expression of this LRR kinase gene depends on NPR1 and is sensitive to benzothiadiazole *S*-methylester (BTH) (Mosher et al., 2006), which suggests that it is a downstream player of the SA signaling pathway.

*TIR-NBS-LRR proteins:* More than 80 TIR-NBS-LRR genes have been identified in the *Arabidopsis* genome (Meyers et al., 2003). A subset of TIR-NBS-LRR genes was also identified as DG3-induced and SR45-suppressed (**Supplementary Table S4**). The proteins for some of them were categorized as cytosolic receptors in ETI signaling. For example, *SUPPRESSOR OF ADR1-L2 1* (*SADR1*) codes for a TIR-NBS-LRR that was recently reported to potentiate plant immunity with or without *EDS1* and to contain pathogen spread (Jacob et al., 2023). *AT3G04220* encodes another TIR-NBS-LRR that contributes to SA-conferred immunity (Lang et al., 2022).

So far, evidence at RNA, metabolite, and genetic levels all led to the same conclusion that the *sr45–1* mutant likely has a higher SAR than Col-0 in the absence of pathogen challenge. It further supports the notion that the *sr45–1* mutant is autoimmune, which is correlated with a smaller plant status and an overall slower growth and development of the mutant plant.

### Did alternative splicing make an impact on the structure and possibly functional levels of any defense gene products?

In addition to its involvement in regulating transcription via the ASAP complex, SR45 is also an RNA-binding protein that interacts with other splicing regulators, such as SCL33 and SR34, and spliceosomal components, such as U1-70K and U2AF, to modulate mRNA splicing (Day et al., 2012; Zhang et al., 2014; Stankovic et al., 2016). In this study, the effect of SR45 and DG3 challenge on alternative splicing using IS events was assessed. Similar numbers of statistically significant IS events were reported in all four contrast groups (**Figure 3A**; **Supplementary Table S5**), ranging from 28 to 41. The genes that code for some of them have been shown to play a role in plant defense, although no significant IS events were identified in SA genes in our results. Two examples are discussed below.

**Figure 5:**
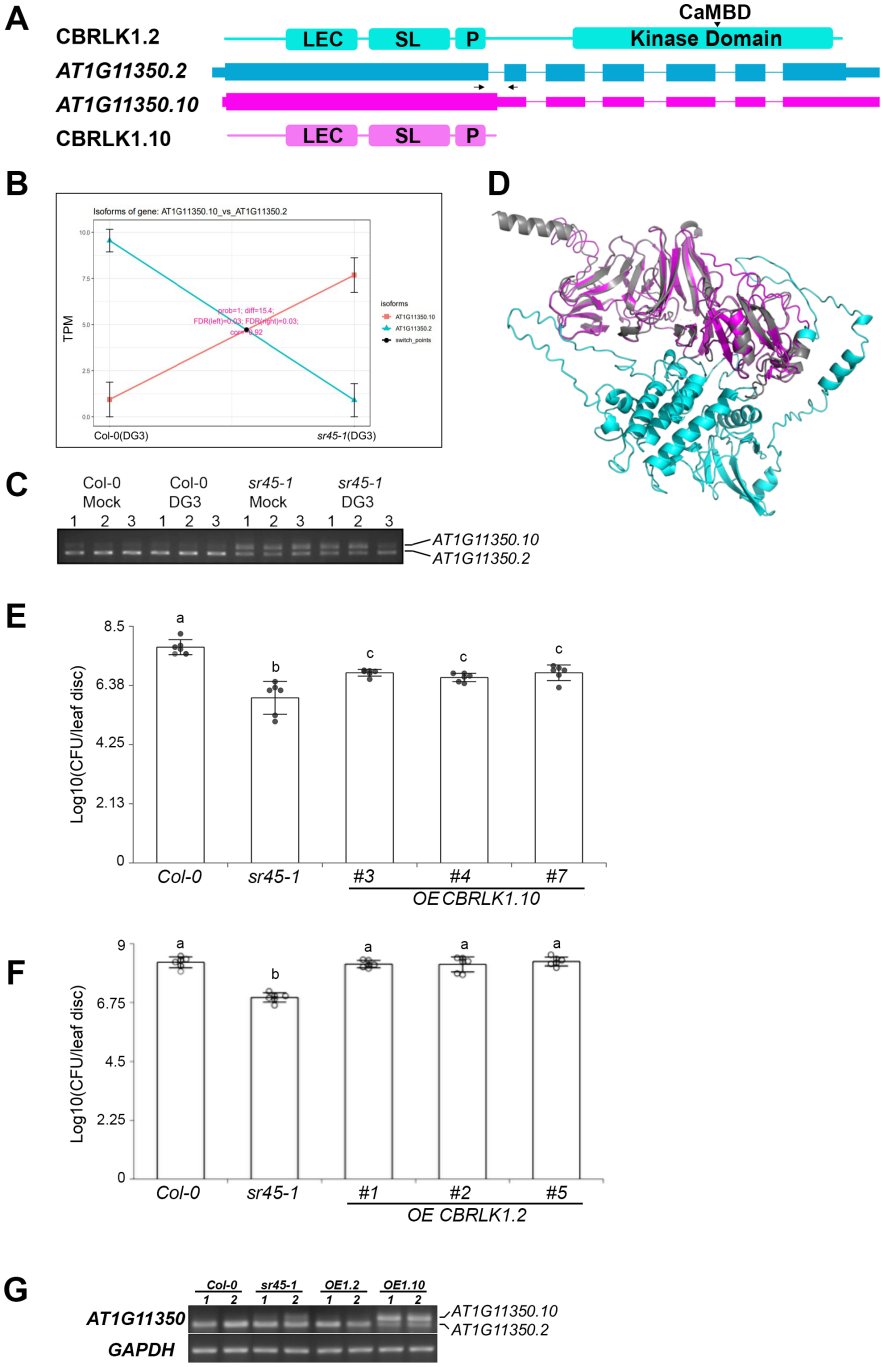
Isoform switch (IS) events in CBRLK1. **(A)** Gene model of two alternatively spliced isoforms of *CBRLK1* gene, *AT1G11350.2* and *AT1G11350.10*, and their corresponding predicted protein domains. LEC, B-lectin domain; SL, S-locus glycoprotein domain; P, plant PAN/APPLE-like domain; CaMBD, calmodulin-binding domain. The position of the pair of primers used in rtPCR is illustrated by a set of arrows. **(B)** Isoform switch plots showing statistically significant switch of the two isoforms illustrated in panel A between Col-0 (DG3) and *sr45-1* (DG3). **(C)** rtPCR showing the expression level of *AT1G11350.2* and *AT1G11350.10* in all samples used in RNA-seq. Numbers 1–3 represent three biological replicates. **(D)** Alignment of predicted protein models for the two CBRLK1 isoforms illustrated in **(A)**. **(E)** Defense response to *Pma*DG3 in CBRLK1.10 overexpression lines, n = 6. Error bars represent standard deviations. One-way ANOVA followed by Tukey's HSD test was used for statistical analysis. Letters a–c represent statistically significant difference (*p* < 0.05). Experiment was repeated for at least two trials. **(F)** Defense response to *Pma*DG3 in CBRLK1.2 overexpression lines, n = 6. Error bars represent standard deviations. One-way ANOVA followed by Tukey's HSD test was used for statistical analysis. Letters a and b represent statistically significant differences (*p* < 0.05). Experiment was repeated for at least two trials. **(G)** rtPCR showing the expression level of *AT1G11350.2* and *AT1G11350.10* in overexpression lines. *OE1.2*, *OE CBRLK1.2*; *OE1.10*, *OE CBRLK1.10*. Two biological replicates were used for each genotype.

*AT1G11350* encodes a LecRLK, calmodulin-binding receptor-like protein kinase (CBRLK1). The full-length protein (CBRLK1.2) is composed of a B-lectin domain (LEC), an S-locus glycoprotein domain (SL), a transmembrane segment (P), and a C-terminal serine/threonine protein kinase domain, with the calmodulin-binding domain located in the middle of the kinase domain (**Figure 5A**). The full-length transcript, *AT1G11350.2*, differs from the alternatively spliced isoform, *AT1G11350.10*, by retention of the first intron. As a result, a pre-mature termination codon (PTC) is introduced in *AT1G11350.10*, resulting in the kinase domain deletion in the protein product, CBRLK1.10 (**Figure 5A**). The abundance of these two isoforms was significantly reversed between WT and *sr45-1* (**Figure 5B**). rtPCR showed that isoform 2 was the dominant isoform in both mock and DG3-treated WT samples, while isoform 10 was the dominant isoform in both mock and DG3-treated *sr45–1* mutant samples (**Figure 5C**), suggesting that this alternative splicing event was controlled by SR45, not DG3. The N-terminus of the two predicted protein models aligned almost perfectly in their LEC, SL, and P domains with a very low root mean square deviation (RMSD) of 0.242 (**Figure 5D**), indicating that CBRLK1.2 may function as an active membrane-bound RLK, whereas CBRLK1.10 may function as a membrane-bound RLP without a kinase activity. Kim et al. 2009b observed that CBRLK1 was localized on the plasma membrane using a GFP tag, and it exhibited a kinase activity *in vitro* (Kim et al., 2009). Genetics studies have shown that CBRLK1.2 was likely a negative regulator of plant defense (Kim et al., 2009a). Based on their studies, one can hypothesize that the truncated protein isoform CBRLK1.10 should have the same plasma membrane localization, but it does not relay the negative regulation due to the lack of the C-terminal kinase domain. Since the RLP isoform (CBRLK1.10) was more abundant in the *sr45–1* mutant, its LEC domain could compete with the RLK isoform (CBRLK1.2) for extracellular ligand binding; however, the RLP lacked the kinase function and could no longer suppress plant immunity as RLK did. We overexpressed CBRLK1.10 in Col-0. The transgenic plants exhibited an intermediate level of resistance to *Pma*DG3 between Col-0 and *sr45-1* (**Figure 5E**). However, when we overexpressed CBRLK1.2 in Col-0, there was no statistical difference in the response to *Pma*DG3 between the transgenic plants and Col-0 (**Figure 5F**). This suggests that CBRLK1.10, not CBRLK1.2, plays a role in the heightened immunity seen in *sr45–1* mutant. The overexpression of each alternatively spliced isoform in these transgenic lines was confirmed using rtPCR (**Figure 5G**).

**Figure 6:**
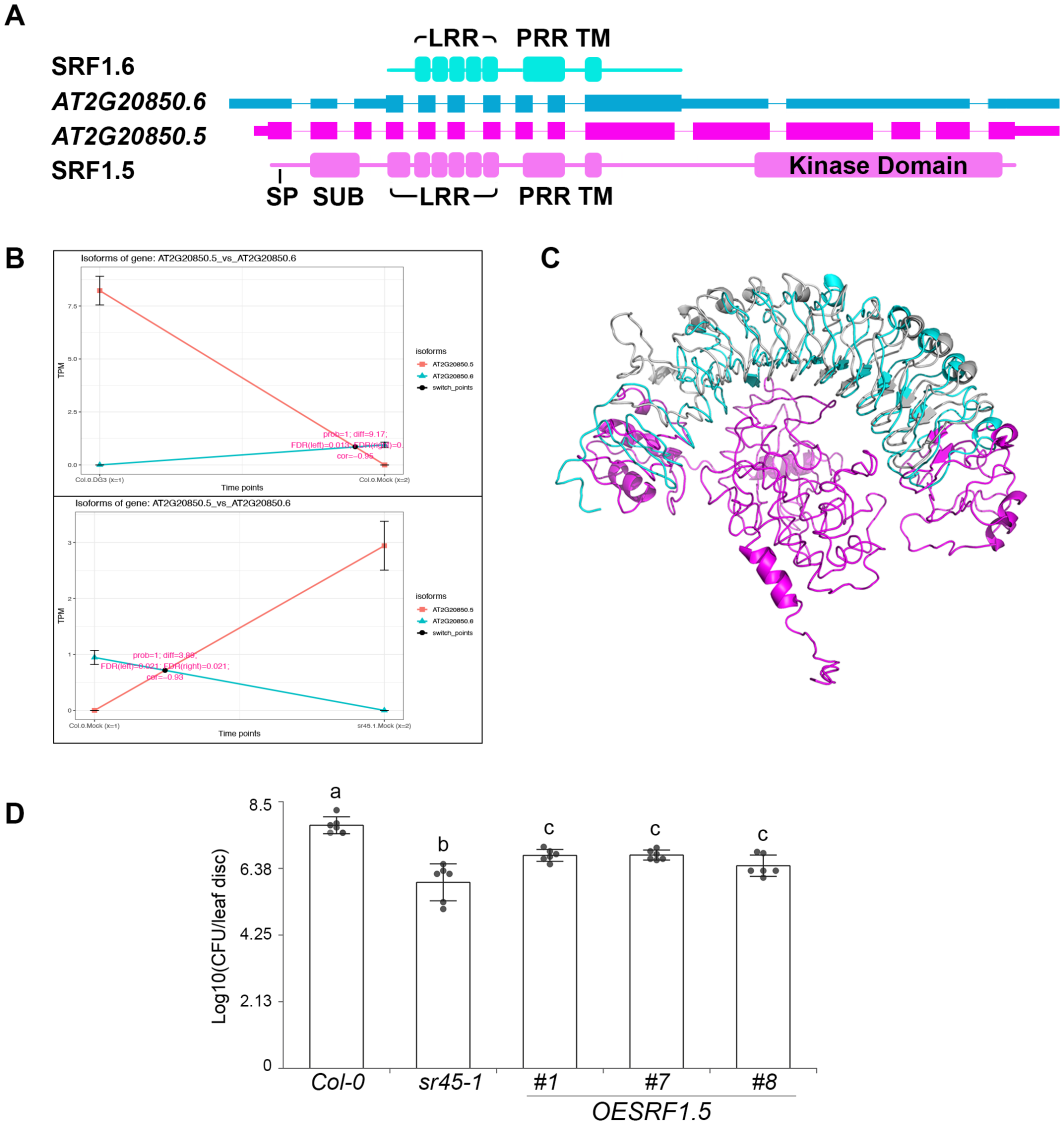
Isoform switch (IS) events in SRF1. **(A)** Gene model of two alternatively spliced isoforms of *SRF1* gene, *AT2G20850.5* and *AT2G20850.6*, and their corresponding predicted protein domains. SP, signal peptide; SUB, SUB domain; LRR, leucine-rich repeat; PRR, proline-rich region; TM, transmembrane domain. **(B)** Isoform switch plots showing statistically significant switch of the two isoforms mentioned in panel **A** between mock and DG3 treatment in Col-0 (top) and between Col-0 and *sr45-1* (bottom). **(C)** Alignment of predicted protein models for the two SRF1 isoforms illustrated in panel **(A, D)** Defense response to *Pma*DG3 in SRF1.5 overexpression lines, n = 6. Error bars represent standard deviations. One-way ANOVA followed by Tukey's HSD test was used for statistical analysis. Letters a–c represent statistically significant differences (*p* < 0.05). Experiment was repeated for at least two trials.

*AT2G20850* locus encodes a putative Type-V LRR-RLK, STRUBBELIG-RECEPTOR FAMILY 1 (SRF1). It has the only IS event that is regulated by both DG3 and SR45. Three intron retention events distinguished the two isoforms (*AT2G20850.5* and *AT2G20850.6*). The second intron retention event was identified in *Intron 10* of *AT2G20850.5*. It is the determinant alternative splicing event that rendered a PTC in *AT2G20850.6*. *AT2G20850.5* codes for a full-length LRR-RLK product (SRF1.5) with a signal peptide (SP), a SUB domain, six LRR, a proline-rich region (PRR), a transmembrane (TM) domain, and a C-terminal kinase domain, while *AT2G20850.6* codes for a putative truncated LRR-RLP product (SRF1.6) with only five LRR, one PRR, and one TM domains (**Figure 6A**). IS plots showed a dramatic increase in abundance for the full-length *LRR-RLK* isoform, *AT2G20850.5*, in response to DG3 challenge and *sr45-1*. The truncated *LRR-RLP* isoform, *AT2G20850.6*, is the dominant product in mock-treated WT (**Figure 6B**). Predicted protein models of SRF1.5 and SRF1.6 did not align perfectly (RMSD = 10.673, **Figure 6C**), suggesting that losing one LRR at the N-terminus could affect the protein structure. These two protein isoforms may differ in their binding affinities to the LRR domain, in addition to the C-terminal kinase activity. The predicted full-length LRR-RLK product, SRF1.5, was named as SRF1A in an earlier study, while the N-terminus of SRF1.6 differs slightly from the SRF1B product (Eyuboglu et al., 2007). Interestingly, this study reported that an overexpression of SRF1.6/SRF1B gave no obvious phenotype, while an overexpression of SRF1.5/SRF1A led to seedling lethality (Eyuboglu et al., 2007). We were successful in obtaining viable transgenic plants overexpressing SRF1.5/SRF1A in WT, and they exhibited an intermediate level of immunity between WT and *sr45-1* (**Figure 6D**), suggesting that SRF1.5 also plays a defense role in *sr45–1* and in response to DG3 challenge. Due to the low expression level of *AT2G20850.6*, we were not able to clone it. No transgenic plants were made to study their effect on plant immunity.

## Discussion

Upon pathogen challenge, plants defend themselves by quickly shifting RNA metabolism to a battle mode, where genes promoting or suppressing immunity could be differentially regulated. SR45 and its homolog RNPS1 are known regulators for multiple steps in the RNA metabolism process, including transcription, splicing, and nonsense-mediated decay (Xing et al., 2015; Questa et al., 2016; Zhang et al., 2017; Mabin et al., 2018). In this study, we discovered that SR45 preferentially suppresses a large subset of pathogen-induced defense genes either at the steady state level or via alternative splicing. Expressing these defense genes can be energy-consuming. By keeping it low when not needed, SR45 could successfully redirect energy toward plant growth and development. The various phenotypes of the *sr45–1* mutant are likely due to the diverse pool of genes that SR45 regulates either directly or indirectly during transcription, splicing, and RNA quality control. In our previous study, we discovered that SR45 associates with transcripts for several splicing factors as well as many transcripts with downstream functions (Zhang et al., 2017). This suggests that SR45, as an upstream regulator, probably functions through a cascade of splicing regulation to amplify its impact on RNA diversity. More interestingly, for some of these genes with significant IS events, SR45 seems to associate with their transcripts (**Supplementary Table S5**), suggesting that SR45 likely regulates their pre-mRNA splicing directly. *CBRLK1*, but not *SRF1*, is among these reported SR45-associated transcripts (**Supplementary Table S5**).

The role of SR45 in transcriptional regulation is less well-known than its function in splicing regulation. The ASAP complex recruits HDA19 to cause gene silencing at the *FLC* locus and promote flowering (Questa et al., 2016). The *sr45–1* null mutant accumulates significantly less amount of the other two ASAP component proteins, SAP18 and ACINUS (ACIN), exhibits an abnormally high level of *FLC*, and has a delayed flowering phenotype. Only a minimum of the remaining SAP18 is located in the nucleus without SR45 (Chen et al., 2019). This implies that SR45 is required for the integrity of the nuclear ASAP complex. This scenario is further supported by a recent *in vitro* study that SAP18 can only bind after SR45-ACIN dimerization (Fanara et al., 2024). Additionally, the *hda19* mutant accumulates SA at a higher level and has a higher expression of defense genes and a heightened defense response (Choi et al., 2012). Further evidence showed that HDA19 also directly modifies histones at the promoter region of several of our DG3-induced and SR45-suppressed genes, such as *PR1*, *PR2*, *EDS5*, and *GDG1*, and suppresses their expression (Choi et al., 2012). Thus, it is possible that SR45 functions through the ASAP complex to recruit HDA19 to the promoter of a subset of defense genes to achieve transcriptional silencing and avoid unwanted immune response in the absence of pathogen challenges. This would be an interesting hypothesis to test in future studies.

One common piece of the puzzle that connects to several groups of genes is the list of transcription factor WRKY coding genes among the DG3-induced and SR45-suppressed genes. It is likely that some of them may be responsible for the expression of a large number of the DG3-induced and SR45-suppressed genes since the W-box is found in 76% of the DG3-induced and SR45-suppressed genes, particularly coding for RLPs, RLKs, and some of the TIR-NBS-LRR genes (**Supplementary Table S4**). It is sensible to speculate that some of the WRKY coding genes were somehow derepressed in *sr45-1*/DG3 challenge. Their protein products work in concert with one another to bind to the W-box sequence in the promoter of a large group of defense genes to trigger transcription and mount a massive defense response.

On the role of SR45 as a splicing regulator, multiple alternatively spliced isoform switch events were identified in our study. Both *CBRLK1* and *SRF1* demonstrated that alternative splicing directly affects the structure of their predicted protein products and their associated functions. Both are examples of an RLK-RLP switch between the two alternatively spliced isoforms. In the case of *CBRLK1*, a putative negative regulator RLK product (CBRLK1.2) is the dominant isoform in WT, while the predicted non-functional RLP product (CBRLK1.10) may cause a competition with the RLK product in *sr45–1* to partially lift the immune suppression. In contrast, *SRF1* is predicted to make a non-functional RLP (SRF1.6) in the mock-treated WT, while the putative alternatively spliced functional RLK (SRF1.5) becomes dominant in mock-treated *sr45–1* and DG3-treated WT. It is very likely that more IS events on the list could produce a pair of alternatively spliced products with one functional and the other not or less functional, depending on how alternative splicing affects the domain structure of the protein product, if the alternative transcript escapes NMD. In the case when the alternative transcript is recognized by NMD, there is no protein made from the alternative transcript at all, rendering a situation resembling a knockdown mutant, given that there is a very low level of constitutive transcript made. Furthermore, *SR45* itself is alternatively spliced. The long isoform, SR45.1, has been shown to regulate flower development (Zhang and Mount, 2009) and a variety of abiotic responses in an isoform-specific manner (Carvalho, Carvalho et al., 2010; Albaqami et al., 2019). Here, we report that SR45.1, but not SR45.2, is also the isoform responsible for SR45’s role in immune suppression. This adds to the repertoire of processes that SR45.1 regulates.

## Conclusion

**Figure 7:**
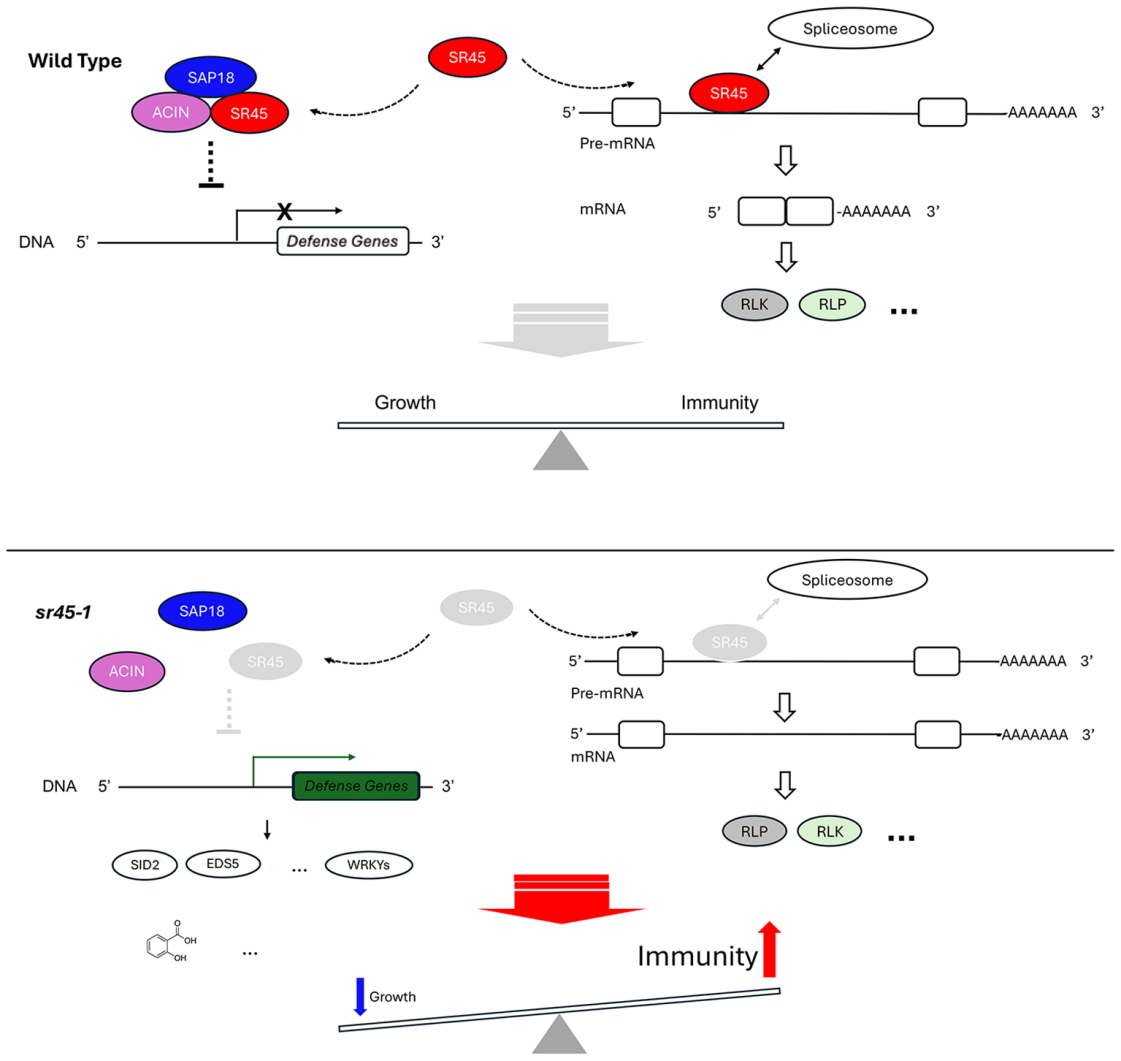
A proposed model illustrating SR45’s dual role in suppressing immune response in *Arabidopsis*. In wild type, SR45 forms ASAP complex with ACIN and SAP18. The ASAP either directly or indirectly suppresses the transcription of defense genes. SR45 is also associated with a subset of pre-mRNAs to recruit spliceosome for intron splicing. There may be other splicing factors involved that are not shown here. Constitutive splicing occurs. The protein products of these constitutively spliced transcripts include RLK and maybe others, such as RLP. Together, SR45 contributes to a growth-immunity balance in *Arabidopsis*. In *sr45-1*, the absence of SR45 prevents the assembly of ASAP complex and the transcriptional suppression of defense genes, including SA biosynthesis genes (for example, *SID2* and *EDS5*) and *WRKY* genes. This leads to the elevated expression of these genes. As a result, the abundance of SA and other defense compounds is increased. Meanwhile, without SR45, spliceosome cannot be recruited to the same pre-mRNAs for normal intron splicing, although there may be a possibility of other splicing factors partially compensating for the loss of SR45, which is not shown here. Alternative splicing takes place at affected loci, resulting in changes in protein structure and possibly function, such as an RLK-RLP or an RLP-RLK switch directly or indirectly. All these transcription-level changes and alternative splicing events eventually lead to a heightened immunity.

In conclusion, our study provides thought-provoking information regarding SR45’s role in plant defense. Possibly, SR45 suppresses plant immunity genes through two main routes: 1) a WRKY-mediated transcriptional regulation network and 2) alternative splicing-induced subsequent domain changes in their corresponding protein products. Multiple levels of evidence strongly suggest that SR45 regulates the level of SAR hormones, Pip, and SA, and their downstream genes to suppress SAR. SA-induced RLP and RLK genes are important targets of SR45 (**Figure 7**). Future work on elucidating the role of the extended SR45-interacting network in immune control via both routes mentioned above will help improve the understanding of how RNA metabolism contributes to a successful immune response in plants.

## Data Availability

The RNA-seq datasets used for this study can be found as a BioProject (PRJNA1260135) in the NCBI Sequence Read Archive (SRA) [http://www.ncbi.nlm.nih.gov/bioproject/1260135]. The datasets presented in this study can be found in online repositories. The names of the repository/repositories and accession number(s) can be found in the article/**Supplementary Material**.

## Supplementary material

Supplementary Figure 1 A functional copy of SR45-mCherry rescued the pleiotropic phenotypes of *sr45-1*. Error bars represent standard deviations. One-Way ANOVA followed by Tukey HSD test was used for statistical analysis. Letters a-d represents statistically significant difference (*p* < 0.05). SR45-mCherry represents *SR45pro::gSR45-mCherry sr45-1. T3-1, T3-3 & T3–9* represent three independent transgenic lines. **(A-D)** SR45-mCherry signal detected in nucleus in different tissues: **(A)** pollen, **(B)** ovule, **(C)** cotyledon and **(D)** root tip. Scale bar = 40 um. Expression level of *SR45* **(E)** and *FLC* **(F)** *. n=3*. *GAPDH* was used housekeeping control. **(G)** Images of plants. **(H)** Days to flower. *n=43* **(I)** Root growth*, n=15* **(J)** defense response to *Pma*DG3. n=6. Experiment was repeated for at least 3 trials.

Supplementary Figure 2 Defense genes are overrepresented in non-tissue specific SR45 differentially regulated genes.

Supplementary Figure 3 Expression profile of SA synthesis genes. Gene TPM values from the 3 biological replicates per sample were used to calculate the average gene TPM for each gene. Error bars represent standard deviations. One-Way ANOVA was used for statistical analysis. Letters a-c represents statistically significant difference (*p* < 0.05).

Supplementary Table 1 Primers used in this study.

Supplementary Table 2 Selected compounds identified with significantly increased abundance from *sr45–1* mutant leaves (s) compared to Col-0 wildtype controls (W).

Supplementary Table 3 A comparison of SR45 differentially regulated genes between leaf (in this study) and inflorescence (Chen et al., 2019).

Supplementary Table 4 A detailed analysis of DG3-induced and SR45-suppressed genes.

Supplementary Table 5 Significant Isoform switch events.

## References

[B1] Albaqami M. Laluk K. Reddy A. S. N. (2019). The Arabidopsis splicing regulator SR45 confers salt tolerance in a splice isoform-dependent manner. Plant Mol. Biol. 100, 379–390. doi: 10.1007/s11103-019-00864-4, PMID: 30968308

[B2] Albert I. Bohm H. Albert M. Feiler C. E. Imkampe J. Wallmeroth N.. (2015). An RLP23-SOBIR1-BAK1 complex mediates NLP-triggered immunity. Nat. Plants 1, 15140. doi: 10.1038/nplants.2015.140, PMID: 27251392

[B3] Ali G. S. Palusa S. G. Golovkin M. Prasad J. Manley J. L. Reddy A. S. (2007). Regulation of plant developmental processes by a novel splicing factor. PloS One 2, e471. doi: 10.1371/journal.pone.0000471, PMID: 17534421 PMC1868597

[B4] Andrews S. (n.d.). FastQC A Quality Control tool for High Throughput Sequence Data. http://www.bioinformatics.babraham.ac.uk/projects/fastqc/

[B5] Bednarek P. Pislewska-Bednarek M. Svatos A. Schneider B. Doubsky J. Mansurova M.. (2009). A glucosinolate metabolism pathway in living plant cells mediates broad-spectrum antifungal defense. Science 323, 101–106. doi: 10.1126/science.1163732, PMID: 19095900

[B6] Bernsdorff F. Doring A. C. Gruner K. Schuck S. Brautigam A. Zeier J. (2016). Pipecolic acid orchestrates plant systemic acquired resistance and defense priming via salicylic acid-dependent and -independent pathways. Plant Cell 28, 102–129. doi: 10.1105/tpc.15.00496, PMID: 26672068 PMC4746677

[B7] Bolger A. M. Lohse M. Usadel B. (2014). Trimmomatic: a flexible trimmer for Illumina sequence data. Bioinformatics 30, 2114–2120. doi: 10.1093/bioinformatics/btu170, PMID: 24695404 PMC4103590

[B8] Carvalho R. F. Carvalho S. D. Duque P. (2010). The plant-specific SR45 protein negatively regulates glucose and ABA signaling during early seedling development in Arabidopsis. Plant Physiol. 154, 772–783. doi: 10.1104/pp.110.155523, PMID: 20699397 PMC2949030

[B9] Chen S. L. Rooney T. J. Hu A. R. Beard H. S. Garrett W. M. Mangalath L. M.. (2019). Quantitative Proteomics Reveals a Role for SERINE/ARGININE-Rich 45 in Regulating RNA Metabolism and Modulating Transcriptional Suppression via the ASAP Complex in Arabidopsis thaliana. Front. Plant Sci. 10, 1116. doi: 10.3389/fpls.2019.01116, PMID: 31608083 PMC6761909

[B10] Choi S. M. Song H. R. Han S. K. Han M. Kim C. Y. Park J.. (2012). HDA19 is required for the repression of salicylic acid biosynthesis and salicylic acid-mediated defense responses in Arabidopsis. Plant J. 71, 135–146. doi: 10.1111/j.1365-313X.2012.04977.x, PMID: 22381007

[B11] Day I. S. Golovkin M. Palusa S. G. Link A. Ali G. S. Thomas J.. (2012). Interactions of SR45, an SR-like protein, with spliceosomal proteins and an intronic sequence: insights into regulated splicing. Plant J. 71, 936–947. doi: 10.1111/j.1365-313X.2012.05042.x, PMID: 22563826

[B12] Denance N. Sanchez-Vallet A. Goffner D. Molina A. (2013). Disease resistance or growth: the role of plant hormones in balancing immune responses and fitness costs. Front. Plant Sci. 4, 155. doi: 10.3389/fpls.2013.00155, PMID: 23745126 PMC3662895

[B13] Ding P. Rekhter D. Ding Y. Feussner K. Busta L. Haroth S.. (2016). Characterization of a pipecolic acid biosynthesis pathway required for systemic acquired resistance. Plant Cell 28, 2603–2615. doi: 10.1105/tpc.16.00486, PMID: 27758894 PMC5134984

[B14] Dodds P. N. Rathjen J. P. (2010). Plant immunity: towards an integrated view of plant-pathogen interactions. Nat. Rev. Genet. 11, 539–548. doi: 10.1038/nrg2812, PMID: 20585331

[B15] Dressano K. Weckwerth P. R. Poretsky E. Takahashi Y. Villarreal C. Shen Z.. (2020). Dynamic regulation of Pep-induced immunity through post-translational control of defence transcript splicing. Nat. Plants 6, 1008–1019. doi: 10.1038/s41477-020-0724-1, PMID: 32690890 PMC7482133

[B16] Durrant W. E. Dong X. (2004). Systemic acquired resistance. Annu. Rev. Phytopathol. 42, 185–209. doi: 10.1146/annurev.phyto.42.040803.140421, PMID: 15283665

[B17] Eyuboglu B. Pfister K. Haberer G. Chevalier D. Fuchs A. Mayer K. F.. (2007). Molecular characterisation of the STRUBBELIG-RECEPTOR FAMILY of genes encoding putative leucine-rich repeat receptor-like kinases in Arabidopsis thaliana. BMC Plant Biol. 7, 16. doi: 10.1186/1471-2229-7-16, PMID: 17397538 PMC1855328

[B18] Fabian M. Gao M. Zhang X. N. Shi J. Vrydagh L. Kim S. H.. (2023). The flowering time regulator FLK controls pathogen defense in Arabidopsis thaliana. Plant Physiol. 191, 2461–2474. doi: 10.1093/plphys/kiad021, PMID: 36662556 PMC10069895

[B19] Fanara S. Schloesser M. Hanikenne M. Motte P. (2022). Altered metal distribution in the sr45–1 Arabidopsis mutant causes developmental defects. Plant J. 110, 1332–1352. doi: 10.1111/tpj.15740, PMID: 35305053

[B20] Fanara S. Schloesser M. Joris M. De Franco S. Vandevenne M. Kerff F.. (2024). The Arabidopsis SR45 splicing factor bridges the splicing machinery and the exon-exon junction complex. J. Exp. Bot. 75 (8), 2280–2298. doi: 10.1093/jxb/erae002, PMID: 38180875

[B21] Gassmann W. Hinsch M. E. Staskawicz B. J. (1999). The Arabidopsis RPS4 bacterial-resistance gene is a member of the TIR-NBS-LRR family of disease-resistance genes. Plant J. 20, 265–277. doi: 10.1046/j.1365-313X.1999.t01-1-00600.x, PMID: 10571887

[B22] Greenberg J. T. Ausubel F. M. (1993). Arabidopsis mutants compromised for the control of cellular damage during pathogenesis and aging. Plant J. 4, 327–341. doi: 10.1046/j.1365-313X.1993.04020327.x, PMID: 8220484

[B23] Guo W. Tzioutziou N. A. Stephen G. Milne I. Calixto C. P. Waugh R.. (2021). 3D RNA-seq: a powerful and flexible tool for rapid and accurate differential expression and alternative splicing analysis of RNA-seq data for biologists. RNA Biol. 18, 1574–1587. doi: 10.1080/15476286.2020.1858253, PMID: 33345702 PMC8594885

[B24] Howard B. E. Hu Q. Babaoglu A. C. Chandra M. Borghi M. Tan X.. (2013). High-throughput RNA sequencing of pseudomonas-infected Arabidopsis reveals hidden transcriptome complexity and novel splice variants. PloS One 8, e74183. doi: 10.1371/journal.pone.0074183, PMID: 24098335 PMC3788074

[B25] Hussain R. M. F. Sheikh A. H. Haider I. Quareshy M. Linthorst H. J. M. (2018). Arabidopsis WRKY50 and TGA transcription factors synergistically activate expression of PR1. Front. Plant Sci. 9, 930. doi: 10.3389/fpls.2018.00930, PMID: 30057584 PMC6053526

[B26] Jacob P. Hige J. Song L. Bayless A. Russ D. Bonardi V.. (2023). Broader functions of TIR domains in Arabidopsis immunity. Proc. Natl. Acad. Sci. U.S.A. 120, e2220921120. doi: 10.1073/pnas.2220921120, PMID: 36893276 PMC10242710

[B27] Jones J. D. Dangl J. L. (2006). The plant immune system. Nature 444, 323–329. doi: 10.1038/nature05286, PMID: 17108957

[B28] Kim H. S. Jung M. S. Lee S. M. Kim K. E. Byun H. Choi M. S.. (2009a). An S-locus receptor-like kinase plays a role as a negative regulator in plant defense responses. Biochem. Biophys. Res. Commun. 381, 424–428. doi: 10.1016/j.bbrc.2009.02.050, PMID: 19222996

[B29] Kim H. S. Jung M. S. Lee K. Kim K. E. Yoo J. H. Kim M. C.. (2009b). An S-locus receptor-like kinase in plasma membrane interacts with calmodulin in Arabidopsis. FEBS Lett. 583, 36–42. doi: 10.1016/j.febslet.2008.11.046, PMID: 19071125

[B30] Kim S. H. Kwon S. I. Saha D. Anyanwu N. C. Gassmann W. (2009). Resistance to the Pseudomonas syringae effector HopA1 is governed by the TIR-NBS-LRR protein RPS6 and is enhanced by mutations in SRFR1. Plant Physiol. 150, 1723–1732. doi: 10.1104/pp.109.139238, PMID: 19525323 PMC2719129

[B31] Kinkema M. Fan W. Dong X. (2000). Nuclear localization of NPR1 is required for activation of PR gene expression. Plant Cell 12, 2339–2350. doi: 10.1105/tpc.12.12.2339, PMID: 11148282 PMC102222

[B32] Kufel J. Diachenko N. Golisz A. (2022). Alternative splicing as a key player in the fine-tuning of the immunity response in Arabidopsis. Mol. Plant Pathol. 23, 1226–1238. doi: 10.1111/mpp.13228, PMID: 35567423 PMC9276941

[B33] Lang J. Genot B. Bigeard J. Colcombet J. (2022). MPK3 and MPK6 control salicylic acid signaling by up-regulating NLR receptors during pattern- and effector-triggered immunity. J. Exp. Bot. 73, 2190–2205. doi: 10.1093/jxb/erab544, PMID: 35032388

[B34] Likić S. Šola I. Ludwig-Müller J. Rusak G. (2014). Involvement of kaempferol in the defence response of virus infected Arabidopsis thaliana. Eur. J. Plant Pathol. 138, 257–271. doi: 10.1007/s10658-013-0326-0

[B35] Mabin J. W. Woodward L. A. Patton R. D. Yi Z. Jia M. Wysocki V. H.. (2018). The Exon Junction Complex Undergoes a Compositional Switch that Alters mRNP Structure and Nonsense-Mediated mRNA Decay Activity. Cell Rep. 25, 2431–2446 e2437. doi: 10.1016/j.celrep.2018.11.046, PMID: 30466796 PMC6328047

[B36] Mayeda A. Badolato J. Kobayashi R. Zhang M. Q. Gardiner E. M. Krainer A. R. (1999). Purification and characterization of human RNPS1: a general activator of pre-mRNA splicing. EMBO J. 18, 4560–4570. doi: 10.1093/emboj/18.16.4560, PMID: 10449421 PMC1171530

[B37] Meyers B. C. Kozik A. Griego A. Kuang H. Michelmore R. W. (2003). Genome-wide analysis of NBS-LRR-encoding genes in Arabidopsis. Plant Cell 15, 809–834. doi: 10.1105/tpc.009308, PMID: 12671079 PMC152331

[B38] Mi H. Ebert D. Muruganujan A. Mills C. Albou L. P. Mushayamaha T.. (2021). PANTHER version 16: a revised family classification, tree-based classification tool, enhancer regions and extensive API. Nucleic Acids Res. 49, D394–D403. doi: 10.1093/nar/gkaa1106, PMID: 33290554 PMC7778891

[B39] Mishra A. K. Baek K. H. (2021). Salicylic acid biosynthesis and metabolism: A divergent pathway for plants and bacteria. Biomolecules 11, 705. doi: 10.3390/biom11050705, PMID: 34065121 PMC8150894

[B40] Mosher R. A. Durrant W. E. Wang D. Song J. Dong X. (2006). A comprehensive structure-function analysis of Arabidopsis SNI1 defines essential regions and transcriptional repressor activity. Plant Cell 18, 1750–1765. doi: 10.1105/tpc.105.039677, PMID: 16766691 PMC1488919

[B41] Onkokesung N. Reichelt M. van Doorn A. Schuurink R. C. van Loon J. J. Dicke M. (2014). Modulation of flavonoid metabolites in Arabidopsis thaliana through overexpression of the MYB75 transcription factor: role of kaempferol-3,7-dirhamnoside in resistance to the specialist insect herbivore Pieris brassicae. J. Exp. Bot. 65, 2203–2217. doi: 10.1093/jxb/eru096, PMID: 24619996 PMC3991749

[B42] Patro R. Duggal G. Love M. I. Irizarry R. A. Kingsford C. (2017). Salmon provides fast and bias-aware quantification of transcript expression. Nat. Methods 14, 417–419. doi: 10.1038/nmeth.4197, PMID: 28263959 PMC5600148

[B43] Questa J. I. Song J. Geraldo N. An H. Dean C. (2016). Arabidopsis transcriptional repressor VAL1 triggers Polycomb silencing at FLC during vernalization. Science 353, 485–488. doi: 10.1126/science.aaf7354, PMID: 27471304

[B44] Savary S. Willocquet L. Pethybridge S. J. Esker P. McRoberts N. Nelson A. (2019). The global burden of pathogens and pests on major food crops. Nat. Ecol. Evol. 3, 430–439. doi: 10.1038/s41559-018-0793-y, PMID: 30718852

[B45] Schornack S. Ballvora A. Gurlebeck D. Peart J. Baulcombe D. Ganal M.. (2004). The tomato resistance protein Bs4 is a predicted non-nuclear TIR-NB-LRR protein that mediates defense responses to severely truncated derivatives of AvrBs4 and overexpressed AvrBs3. Plant J. 37, 46–60. doi: 10.1046/j.1365-313X.2003.01937.x, PMID: 14675431

[B46] Schwerk C. Prasad J. Degenhardt K. Erdjument-Bromage H. White E. Tempst P.. (2003). ASAP, a novel protein complex involved in RNA processing and apoptosis. Mol. Cell Biol. 23, 2981–2990. doi: 10.1128/MCB.23.8.2981-2990.2003, PMID: 12665594 PMC152566

[B47] Singh B. K. Delgado-Baquerizo M. Egidi E. Guirado E. Leach J. E. Liu H.. (2023). Climate change impacts on plant pathogens, food security and paths forward. Nat. Rev. Microbiol. 21, 640–656. doi: 10.1038/s41579-023-00900-7, PMID: 37131070 PMC10153038

[B48] Stankovic N. Schloesser M. Joris M. Sauvage E. Hanikenne M. Motte P. (2016). Dynamic distribution and interaction of the arabidopsis SRSF1 subfamily splicing factors. Plant Physiol. 170, 1000–1013. doi: 10.1104/pp.15.01338, PMID: 26697894 PMC4734559

[B49] Steidele C. E. Stam R. (2021). Multi-omics approach highlights differences between RLP classes in Arabidopsis thaliana. BMC Genomics 22, 557. doi: 10.1186/s12864-021-07855-0, PMID: 34284718 PMC8290556

[B50] Xing D. Wang Y. Hamilton M. Ben-Hur A. Reddy A. S. (2015). Transcriptome-wide identification of RNA targets of arabidopsis SERINE/ARGININE-RICH45 uncovers the unexpected roles of this RNA binding protein in RNA processing. Plant Cell 27, 3294–3308. doi: 10.1105/tpc.15.00641, PMID: 26603559 PMC4707455

[B51] Xu F. Xu S. Wiermer M. Zhang Y. Li X. (2012). The cyclin L homolog MOS12 and the MOS4-associated complex are required for the proper splicing of plant resistance genes. Plant J. 70, 916–928. doi: 10.1111/j.1365-313X.2012.04906.x, PMID: 22248079

[B52] Yang S. Gao M. Xu C. Gao J. Deshpande S. Lin S.. (2008). Alfalfa benefits from Medicago truncatula: the RCT1 gene from M. truncatula confers broad-spectrum resistance to anthracnose in alfalfa. Proc. Natl. Acad. Sci. U.S.A. 105, 12164–12169. doi: 10.1073/pnas.0802518105, PMID: 18719113 PMC2527883

[B53] Yang S. Tang F. Zhu H. (2014). Alternative splicing in plant immunity. Int. J. Mol. Sci. 15, 10424–10445. doi: 10.3390/ijms150610424, PMID: 24918296 PMC4100160

[B54] Yang J. Yan R. Roy A. Xu D. Poisson J. Zhang Y. (2015). The I-TASSER Suite: protein structure and function prediction. Nat. Methods 12, 7–8. doi: 10.1038/nmeth.3213, PMID: 25549265 PMC4428668

[B55] Zhang R. Kuo R. Coulter M. Calixto C. P. G. Entizne J. C. Guo W.. (2022). A high-resolution single-molecule sequencing-based Arabidopsis transcriptome using novel methods of Iso-seq analysis. Genome Biol. 23, 149. doi: 10.1186/s13059-022-02711-0, PMID: 35799267 PMC9264592

[B56] Zhang X. N. Mo C. Garrett W. M. Cooper B. (2014). Phosphothreonine 218 is required for the function of SR45.1 in regulating flower petal development in Arabidopsis. Plant Signal Behav. 9, e29134. doi: 10.4161/psb.29134, PMID: 25763493 PMC4203572

[B57] Zhang X. N. Mount S. M. (2009). Two alternatively spliced isoforms of the Arabidopsis SR45 protein have distinct roles during normal plant development. Plant Physiol. 150, 1450–1458. doi: 10.1104/pp.109.138180, PMID: 19403727 PMC2705014

[B58] Zhang X. N. Shi Y. Powers J. J. Gowda N. B. Zhang C. Ibrahim H. M. M.. (2017). Transcriptome analyses reveal SR45 to be a neutral splicing regulator and a suppressor of innate immunity in Arabidopsis thaliana. BMC Genomics 18, 772. doi: 10.1186/s12864-017-4183-7, PMID: 29020934 PMC5637254

[B59] Zhou M. Wang W. Karapetyan S. Mwimba M. Marques J. Buchler N. E.. (2015). Redox rhythm reinforces the circadian clock to gate immune response. Nature 523, 472–476. doi: 10.1038/nature14449, PMID: 26098366 PMC4526266

[B60] Zhu Z. Xu F. Zhang Y. Cheng Y. T. Wiermer M. Li X.. (2010). Arabidopsis resistance protein SNC1 activates immune responses through association with a transcriptional corepressor. Proc. Natl. Acad. Sci. U.S.A. 107, 13960–13965. doi: 10.1073/pnas.1002828107, PMID: 20647385 PMC2922275

