# Supplementary figures and images for: A splicing regulator, SR45, suppresses plant immunity by regulating salicylic acid pathway in Arabidopsis thaliana
Source: Front Plant Sci. 2025 Oct 31;16:1704701. doi: 10.3389/fpls.2025.1704701 (PMC12615492; doi:10.3389/fpls.2025.1704701)

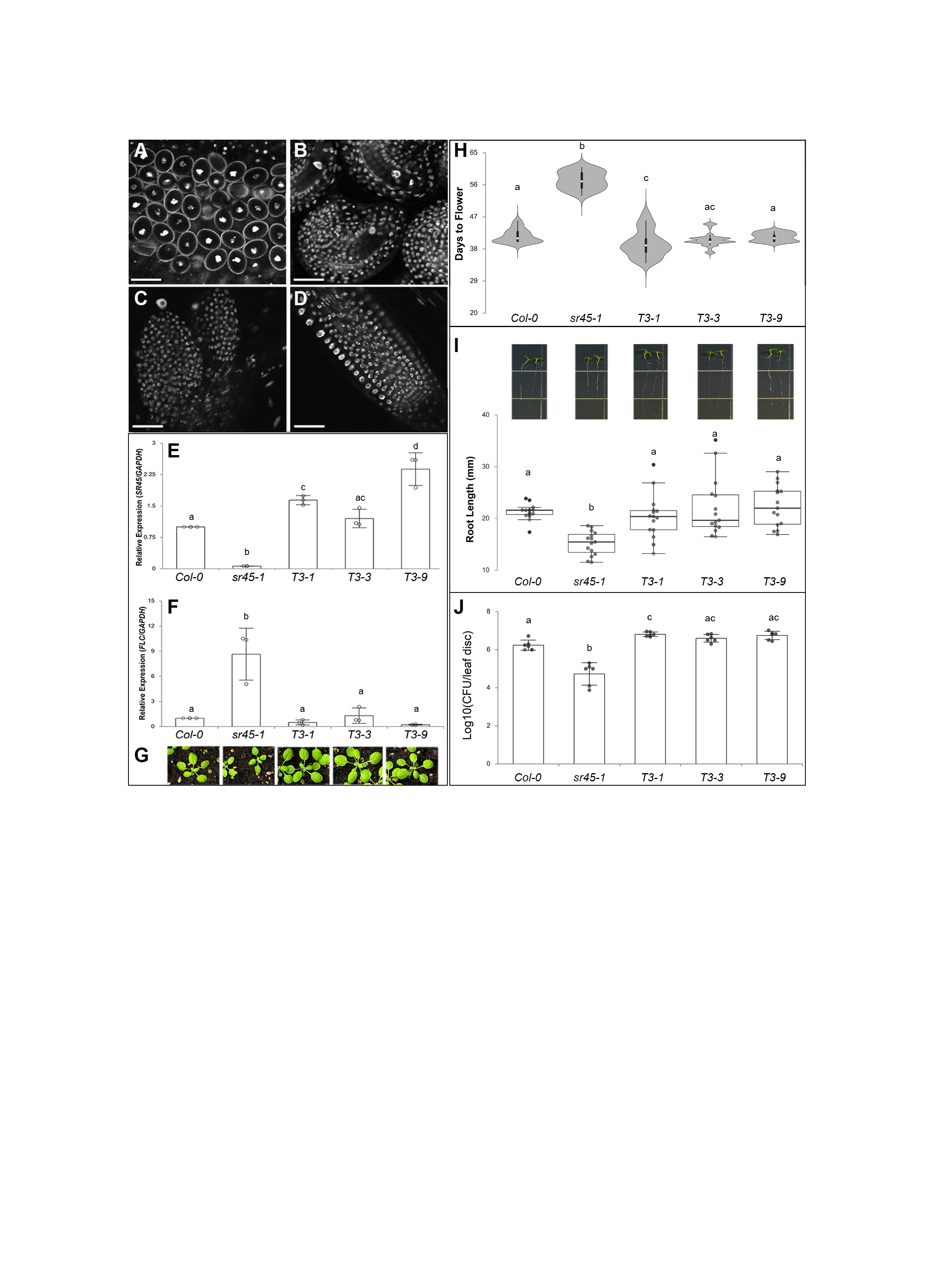

Supplement: Supplementary Figure 1 — A functional copy of SR45-mCherry rescued the pleiotropic phenotypes of sr45-1. Error bars represent standard deviations. One-Way ANOVA followed by Tukey HSD test was used for statistical analysis. Letters a-d represents statistically significant difference (p < 0.05). SR45-mCherry represents SR45pro::gSR45-mCherry sr45-1. T3-1, T3-3 & T3–9 represent three independent transgenic lines. (A-D) SR45-mCherry signal detected in nucleus in different tissues: (A) pollen, (B) ovule, (C) cotyledon and (D) root tip. Scale bar = 40 um. Expression level of SR45 (E) and FLC (F) . n=3. GAPDH was used housekeeping control. (G) Images of plants. (H) Days to flower. n=43 (I) Root growth, n=15 (J) defense response to PmaDG3. n=6. Experiment was repeated for at least 3 trials. [file Image1.tif]

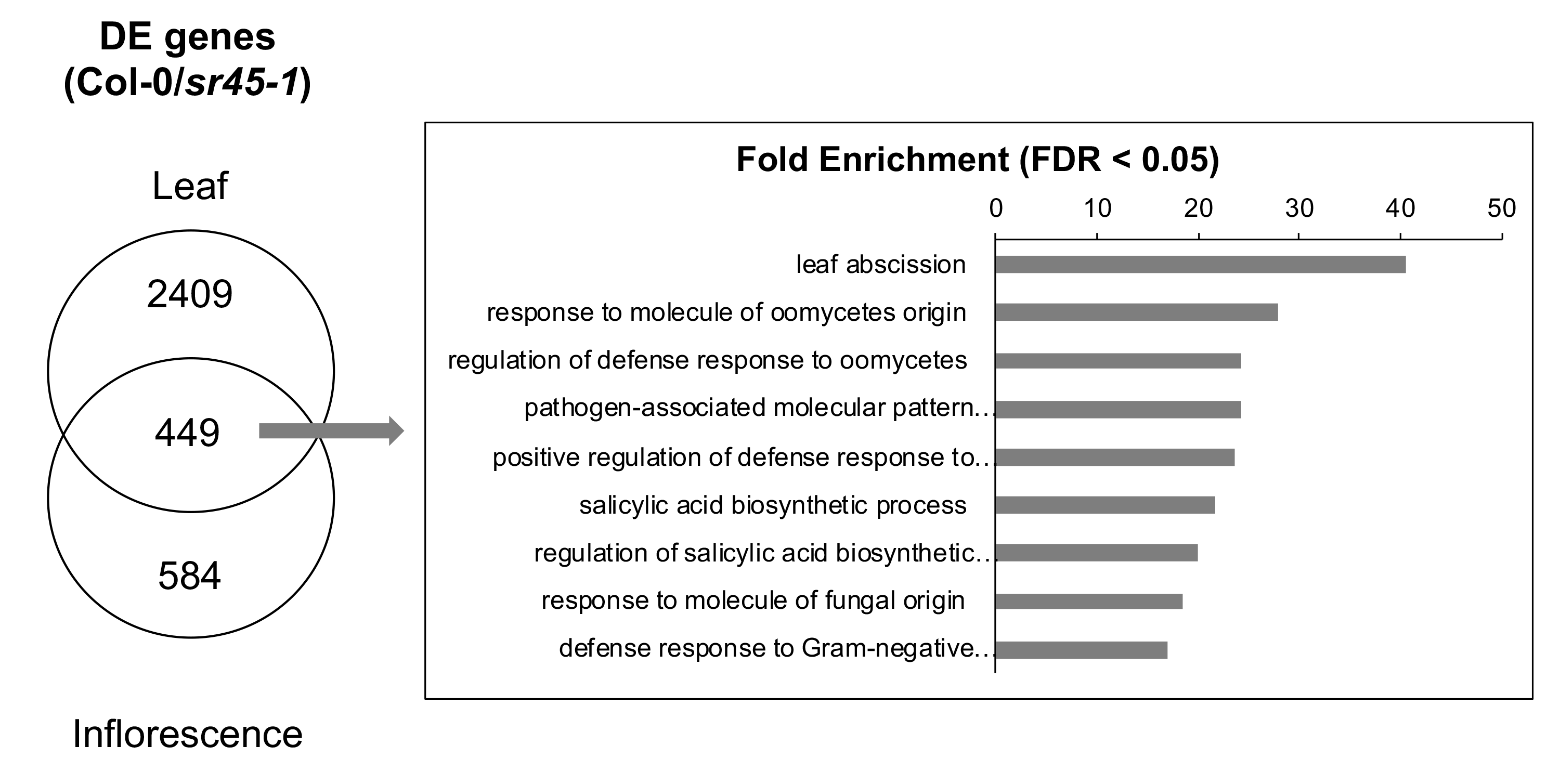

Supplement: Supplementary Figure 2 — Defense genes are overrepresented in non-tissue specific SR45 differentially regulated genes. [file Image2.tif]

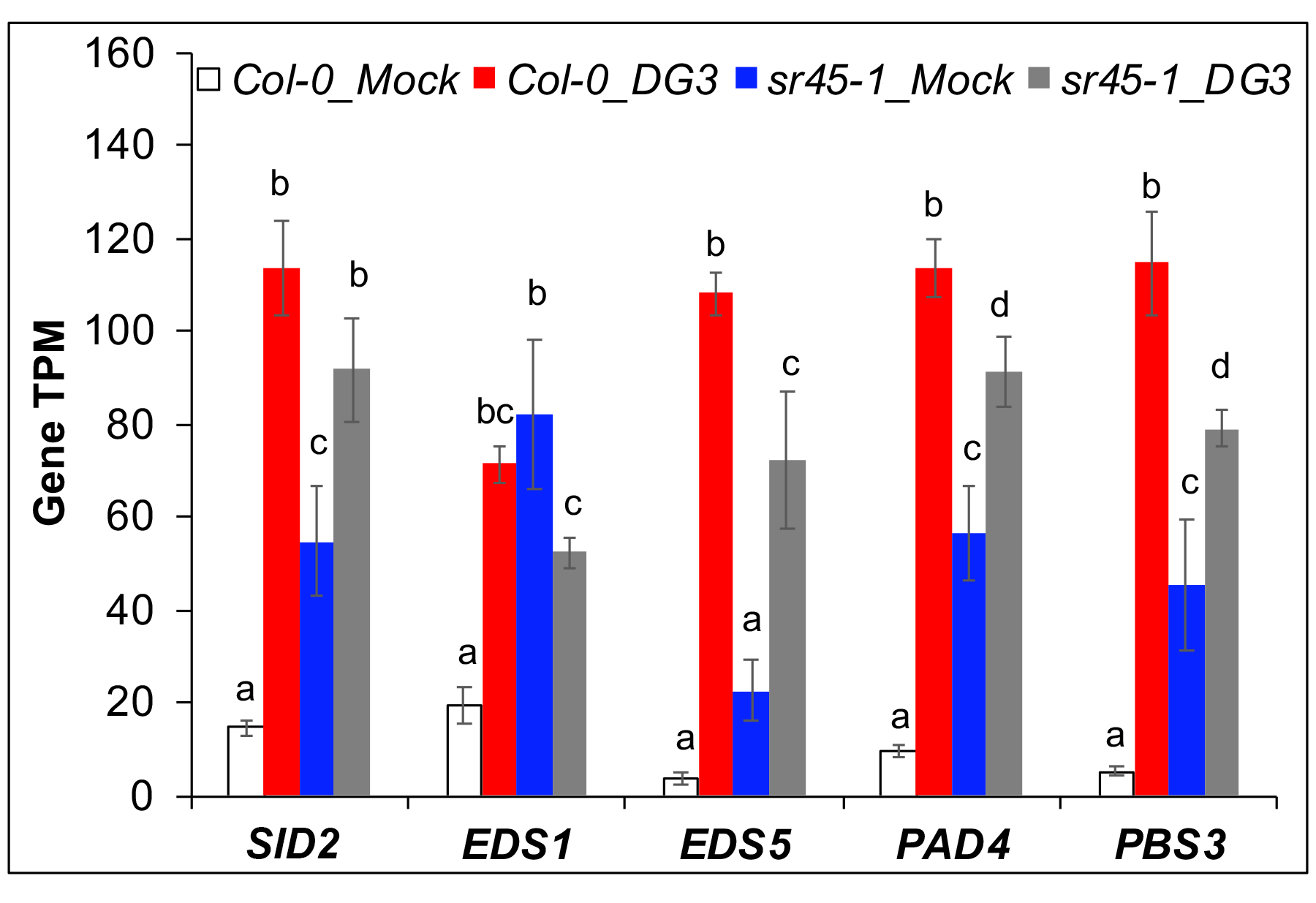

Supplement: Supplementary Figure 3 — Expression profile of SA synthesis genes. Gene TPM values from the 3 biological replicates per sample were used to calculate the average gene TPM for each gene. Error bars represent standard deviations. One-Way ANOVA was used for statistical analysis. Letters a-c represents statistically significant difference (p < 0.05). [file Image3.tif]
